# MEPO-ML: a robust graph attention network model for rapid generation of partial atomic charges in metal-organic frameworks

**DOI:** 10.1038/s41524-024-01413-4

**Published:** 2024-09-18

**Authors:** Jun Luo, Omar Ben Said, Peigen Xie, Marco Gibaldi, Jake Burner, Cécile Pereira, Tom K. Woo

**Affiliations:** 1 Department of Chemistry and Biomolecular Science, University of Ottawa, 10 Marie Curie Private, Ottawa, K1N 6N5 Canada; 2 TotalEnergies OneTech SE, Palaiseau, France

**Keywords:** Metal-organic frameworks, Metal-organic frameworks, Atomistic models

## Abstract

Accurate computation of the gas adsorption properties of MOFs is usually bottlenecked by the DFT calculations required to generate partial atomic charges. Therefore, large virtual screenings of MOFs often use the QEq method which is rapid, but of limited accuracy. Recently, machine learning (ML) models have been trained to generate charges in much better agreement with DFT-derived charges compared to the QEq models. Previous ML charge models for MOFs have all used training sets with less than 3000 MOFs obtained from the CoRE MOF database, which has recently been shown to have high structural error rates. In this work, we developed a graph attention network model for predicting DFT-derived charges in MOFs where the model was developed with the ARC-MOF database that contains 279,632 MOFs and over 40 million charges. This model, which we call *MEPO-ML*, predicts charges with a mean absolute error of 0.025e on our test set of over 27 K MOFs. Other ML models reported in the literature were also trained using the same dataset and descriptors, and MEPO-ML was shown to give the lowest errors. The gas adsorption properties evaluated using MEPO-ML charges are found to be in significantly better agreement with the reference DFT-derived charges compared to the empirical charges, for both polar and non-polar gases. Using only a single CPU core on our benchmark computer, MEPO-ML charges can be generated in less than two seconds on average (including all computations required to apply the model) for MOFs in the test set of 27 K MOFs.

## Introduction

Metal-organic framework (MOF) materials have attracted significant attention as next-generation solid sorbents for gas separation and storage applications such as CO_2_ capture. For example, the MOF CALF-20 is being used in an adsorption-based process that is being deployed at an industrial scale to remove CO_2_ from cement-making flues and is now being synthesized in over 1.5-ton batches^1,2^. The attractive feature of MOFs is their tunability which arises from the seemingly infinite number of structural building units (SBUs) that can combine in thousands of different network topologies to form structures with a daunting variety of geometric and chemical features. It is estimated that over 100,000 MOFs have been synthesized and crystallographically characterized, with new materials being continually reported^3^. Additionally, various computer algorithms^4–6^ have been developed to construct hypothetical MOF (hMOF) structures where millions have been used in the curation of various databases^7–10^.

High throughput virtual screening studies have been performed on databases of both experimental and hypothetical MOFs to identify high-performing materials for a variety of applications. For gas separation and storage applications, a material’s performance is often evaluated in terms of its uptake capacity and selectivity. These properties can be computed via atomistic grand-canonical Monte Carlo (GCMC) simulations. Here the guest-host interaction energies are commonly approximated by atom-pair based van der Waals potentials (e.g., Lennard-Jones potentials), and Coulomb potentials using partial atomic charges. While the parameters used in the van der Waals potentials are largely considered to be transferable between different MOFs, the partial atomic charges used for the Coulomb interactions are highly dependent on the chemical environment of the atoms and therefore need to be re-computed for each individual material. There are many types of partial atomic charges one can use since they are not a physical observable. However, the so-called electrostatic potential-fitted (ESP) charges are the most accurate in computing the electrostatic energy of a chemical system in classical atomistic simulations. These ESP charges are derived from a density functional theory (DFT) calculation of the system and fit such that the electrostatic potential due to the point charges best reproduces the electrostatic potential from the DFT calculation on a set of grid points surrounding the atoms in the system. For simulations of molecular systems, ESP charges have been used for decades, with the RESP^11,12^ and CHELPG^13^ methods being particularly popular. The REPEAT method^14^ was the first ESP charge method that could derive charges from periodic DFT calculations. Later, Manz and Sholl developed another ESP based method, called the DDEC method^15^, that is popular for atomistic simulations of MOFs. While ESP charges are considered accurate in that they reproduce the DFT-derived electrostatic potential, they require a compute-intensive first principles DFT calculation to be performed on the system. For MOFs containing thousands of atoms in the unit cell, a periodic DFT calculation can take hundreds of CPU hours, which is not practical when screening large MOF databases containing tens of thousands of structures or more. To overcome this bottleneck, rapid empirical methods based on physical charge-sharing models were first employed to compute the atomic charges in high throughput screening studies^16,17^. The most popular of these methods are the charge equilibration (QEq) method^18^ and the more generalized split charge equilibration (SQE) method^19^ both of whose charges heavily depend on the parameterization. Kadantsev et al. developed a popular MOF-optimized parameterization of the QEq method called MEPO-QEq^20^ that was fit to reproduce REPEAT charges trained on a set of 543 hMOFs. This set of hMOFs included 4 common metallic nodes, 52 different organic linkers, 17 different functional groups and 11 atom types. Collins et al. later developed a MOF-optimized parameterization for the SQE method, called SQE-MEPO^21^. With a diverse training set of 559 hMOFs and 45 hypothetical porous polymer networks (PPNs) and a separate test set of 520 MOFs and 65 PPNs, a total of 91 parameters were optimized for 17 elements and 31 bond types. Due to the limitations in the physical models, the charge equilibration methods may produce distributions of charges that can differ significantly from the ESP charges, particularly for some metal elements^21^.

Machine learning (ML) models have become increasingly popular for predicting compute-intensive chemical properties, including models specifically for predicting ESP charges^22–24^. In general, these ML models are trained to map descriptors that encode an atom’s chemical environment in a structure to its corresponding ESP charge. For predicting partial atomic charges in MOFs, several ML models have been developed that can predict charges for all atoms in a system in seconds while showing much better agreement with the reference DFT-derived ESP charges than empirical charges^25–27^. Curating or selecting datasets for training is usually the first step in developing ML models. Here, the Computation-Ready, Experimental (CoRE) databases^28,29^ have been exclusively used for developing machine learning models for predicting partial atomic charges in MOFs. The CoRE databases are collections of experimentally characterized MOF structures taken from the Cambridge Structure Database (CSD)^30^ that have been prepared for use in computational simulations. The first version of the CoRE database^28^ was published in 2014 and contained close to 4.7 K structures, while an updated version^29^ published in 2019 contains over 16 K MOF structures.

Using different subsets of the CoRE databases, researchers have built ML models for predicting partial atomic charges in MOFs using various atomic descriptors and ML architectures. Korolev et al.^25^ trained a gradient boosting decision tree regressor (GBDTR) for predicting charges in MOFs using DDEC charges computed for 2,932 MOFs from the CoRE 2014 database. This model used a collection of atomic descriptors, including (i) intrinsic elemental properties of the atom (e.g., atomic number, covalent radius, and electronegativity), (ii) structural descriptors of the atom, (e.g., coordination number and Voronoi fingerprints), as well as (iii) distance-based descriptors (e.g., atom-centered symmetry functions, ACSFs^31^, and AGNI fingerprints^32^). Korolev et al. reported a mean absolute error (MAE) of 0.010 e compared to the reference DDEC charges from their test set. Using DDEC charges computed on 2,974 MOFs from the CoRE 2019 database, Kancharlapalli et al.^26^ developed a random forest regressor (RFR) to predict partial atomic charges in MOFs. They used a set of descriptors consisting of two elemental properties (electronegativity and first ionization energy) and five connectivity-based descriptors based on chemical bonding: coordination number, average bond distance, average electronegativity of the neighboring bonded atoms, and average first ionization energies in the first and second coordination shells. Kancharlapalli et al. reported an MAE of 0.019 e on their test set.

Both GBDTR and RFR methods described above are decision tree-based models that learn chemical bonding information via structural descriptors. These models are only aware of chemical environments of discrete atoms during training and inference. Graph neural networks (GNNs), on the other hand, can learn chemical bonding information for each atom directly from the chemical graph. Molecules and periodic materials can be abstracted to chemical graphs by encoding the atoms as nodes and the bonds as edges, where additional descriptors can be assigned to the nodes (e.g., atomic number and electronegativity) and edges (e.g., bond length and bond order). Raza et al.^27^ trained a gated graph neural network (GGNN) to predict DDEC charges in MOFs, with only atomic numbers assigned to the nodes of the graph. The “message passing” mechanism in the GGNN exchanges the hidden embeddings of the atoms across the chemical bonds in each layer of the GGNN, therefore allowing the model to incorporate information on the atom of interest as well as the bonded atoms during training and inference. Raza et al. used DDEC charges from 2266 MOFs from the CoRE 2014 database for developing their GGNN model and reported an MAE of 0.025 e on their test set.

All three abovementioned ML models achieved test set MAEs of 0.025 e or better against the reference DDEC charges, which is a significant improvement upon the empirical charge generation methods, whose MAEs are usually on the order of 0.1 e. These works have clearly demonstrated the algorithmic advantages of ML models over the physical charge-sharing models (QEq, SQE, etc.). However, in all three studies, less than 3000 MOFs were used to develop the ML models and a detailed diversity analysis of the SBU chemistry was not performed on the training sets. Moreover, there have been progressively more reports pointing out the existence of severe structural errors in the CoRE databases^33–35^. Most alarmingly, a recent manual inspection of the CoRE databases has shown that almost 50% of the structures in these databases have structural errors, such as missing protons, or incorrectly assigned framework charges^34^. Nearly all of these structural errors are manifested in metal oxidation state assignments that are either impossible, never before observed or extremely rare in stable compounds. Such erroneous structures are analogous to molecules containing hexavalent carbon atoms in a drug candidate database – they are not chemically sensible and therefore not synthetically accessible. Indeed, a manual inspection of all structures in the datasets used to develop the three previously mentioned ML charge models reveals that they still contain 16% to 22% erroneous MOF structures. With such severe structural errors likely making up significant portions of the relatively small training sets used to develop the three ML partial charge models for MOFs, the fidelity and transferability of these models may be questioned.

In this work, we present an ML charge prediction model for MOFs that attempts to address the shortcomings of previous ML charge models, which we refer to as the MEPO-ML (Mof Electrostatic POtential-Machine Learned) model. The MEPO-ML model was developed with the ARC-MOF database^9^ that contains 279,632 MOFs for which DFT-derived REPEAT charges have been calculated. This database contains approximately two orders of magnitude more MOF structures than the datasets used to develop previous models, which translates to much more diverse atomic environments for ML models to learn from. Moreover, the ARC-MOF database was screened for the kind of structural errors found in the CoRE databases; we estimate that the structural error rate in ARC-MOF is less than 5% compared to ~50% found in CoRE. In terms of the ML architecture, MEPO-ML was developed with a more advanced graph attention network (GAT). The GAT architecture adds an “attention mechanism” to a generic message passing graph-neutral network, such that the model also learns to weigh the messages from different neighboring nodes. This is analogous to the concept of electronegativity in chemistry, where the fluorine atom would attract more electrons compared to the hydrogen atom in an F-C-H fragment. Therefore “messages” from the fluorine atom would have more “weight” (or get more “attention”) than from the hydrogen atom when passing messages to the carbon. Our GAT-based MEPO-ML model is compared to RFR, GBDTR and GGNN models for charge predictions using the same training and test sets. This is important since it was difficult to compare to previously reported models since different datasets were used in developing each model. We also analyze the descriptor importance and the effects of training set size for each model. Gas adsorption properties from atomistic simulations using MEPO-ML charges are compared against those using the reference DFT-derived REPEAT charges and the empirical SQE-MEPO charges. Lastly, we also benchmark the inference speed of our MEPO-ML model in our charge prediction workflow.

## Results

### Datasets

**Fig. 1:**
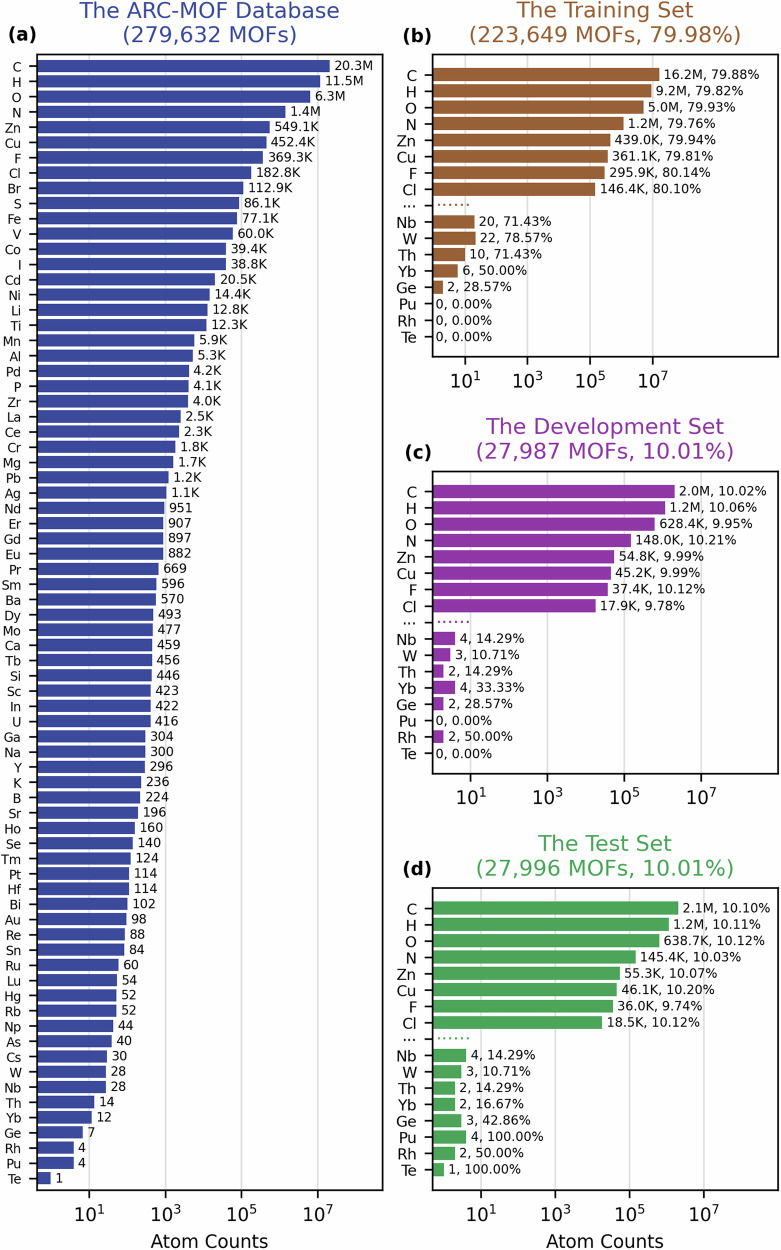
MOF and atom counts in the datasets. Log scale histograms for the atom counts of the datasets used in this work with MOF counts stated in the title of each panel: **a** the atom counts of all 74 elements in the ARC-MOF database; atom counts for the top 8 most popular and rare elements of (**b**) the training set, **c** the development set, **d** the test set. Large values in the bar labels are rounded to millions, M, or thousands, K; the percentage besides the atom count labels indicates the percentage taken from the entire ARC-MOF database.

The 279,632 MOFs of the ARC-MOF database were split into a training, development and test set in a 80:10:10 ratio. Normally, this would be done by random splitting, however, as shown in Fig. 1a, the population of elements in the ARC-MOF database varies by several orders of magnitude. For example, there are more than 20 million carbon atoms in the entire database whereas there are only 28 niobium atoms. To avoid the exclusion of certain elements by random splitting due to their rarity, a custom splitting scheme was used as described in the Methods section. Figure 1b–d presents the atom counts in the training, development and test sets for selected elements – including the top 8 most abundant elements as well as the top 8 most rare elements. The figures demonstrates that the custom splitting procedure was able to split the whole database into subsets where the atom count by element was maintained in an 80:10:10 ratio for abundant elements (e.g. C, H, O, N, Zn, Cu, F and Cl in Fig. 1b–d). Although the ~40 million atoms could have been individually split into the three sets, it is important to note that in this work, all atoms in a given MOF are put into the same dataset. Thus, for rare elements, such as Nb, W, Th, Yb, and Ge in Fig. 1, the splitting ratio diverges from the target 80:10:10 due to the varying number of atoms in the unit cells of the MOFs containing these elements. For elements of extreme rarity (Rh, Pu, Te), where there are less than 2 MOFs containing these elements in the whole dataset, we prioritized their inclusion in the test or development set, since we are interested in the model’s transferability to elements that are absent in the training set. The exact splits of the datasets used for this work are given Supplementary Table E1 as a separate spreadsheet for reproducibility purpose.

### Performance of the ML Models

**Fig. 2:**
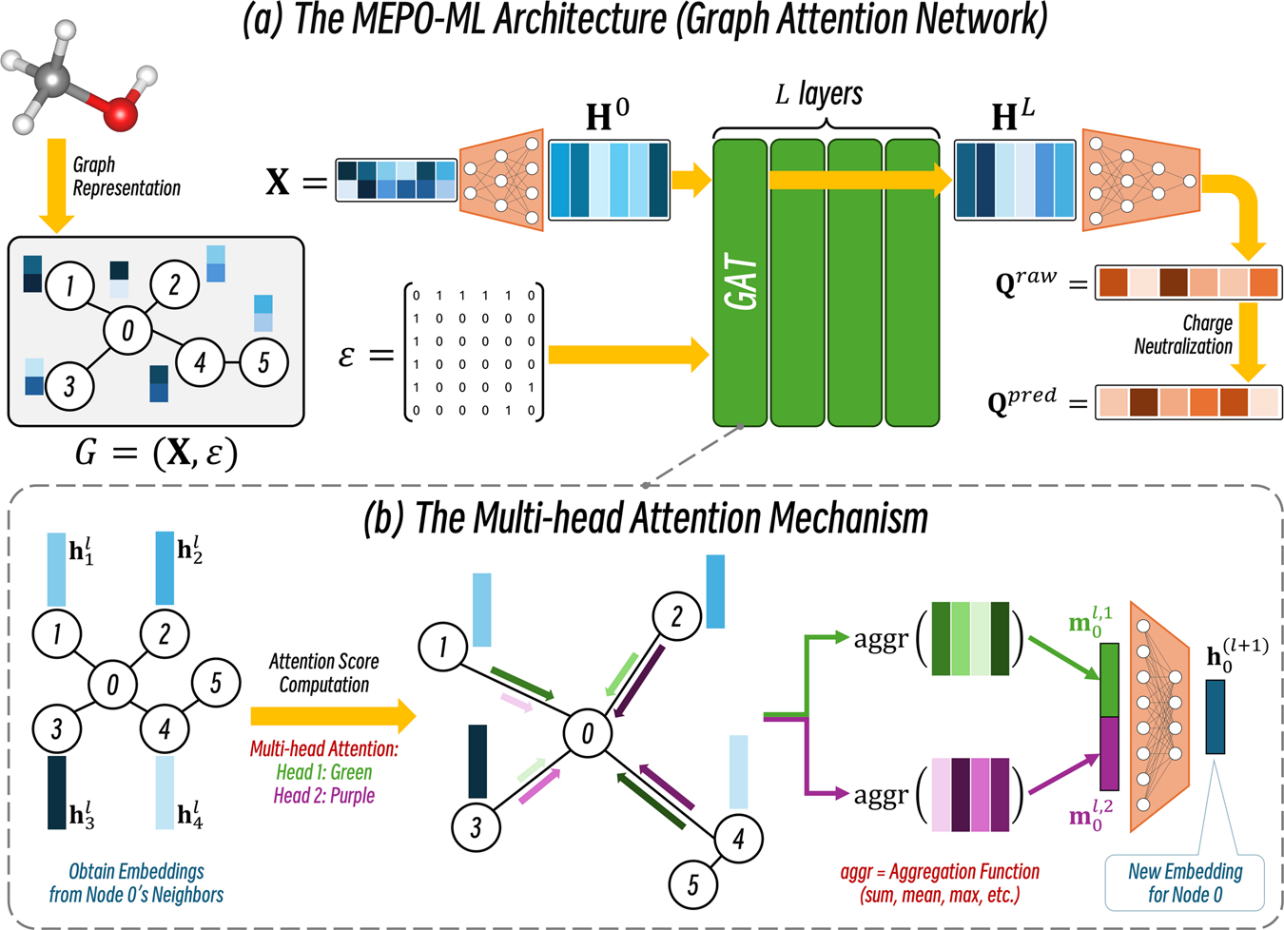
The overall architecture of the MEPO-ML model. **a** The inference workflow of MEPO-ML from converting chemical structures into graphs to charge predictions by the graph attention network (GAT). **b** A more detailed schematic of each graph attention convolution layer and the multi-head attention mechanism used in the GAT employed by MEPO-ML.

**Fig. 3:**
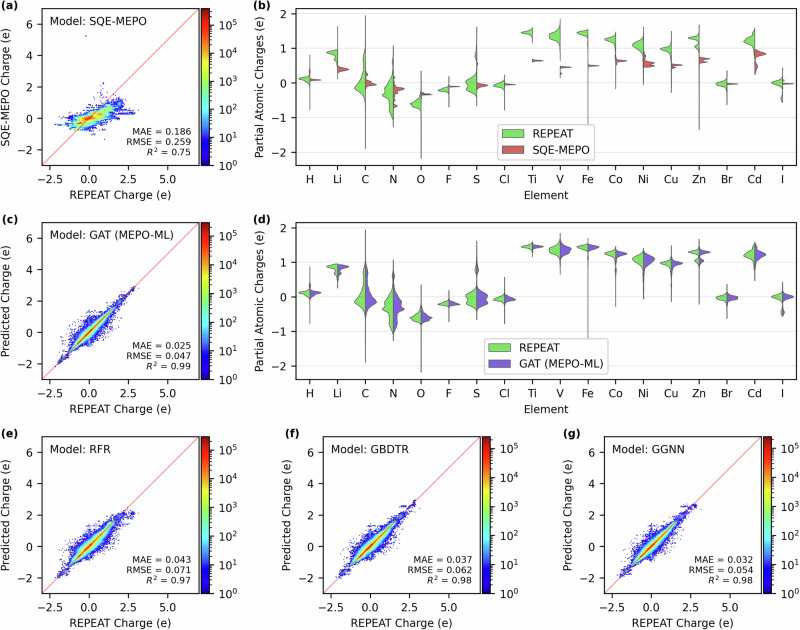
Performance comparisons of different charge generation methods. Parity plots comparing the reference REPEAT charges in the test set to those generated by the (**a**) split charge equilibrium, SQE-MEPO, **c** graph attention network, GAT, **e** random forest regressor, RFR, **f** gradient boosted decision tree regressor, GBDTR, and (**g**) gated graph neural network, GGNN models. All metrics given are computed using the test set. The pale red lines indicates parity. Violin plots comparing the distributions of REPEAT charges to the distributions of predicted charges for the 18 most common elements are given in (**b**) for the SQE-MEPO model and (**d**) for the GAT model. Note that all charges plotted here are following charge neutralization.

Figure 2 illustrates the ML architecture of the graph attention network (GAT) employed by the MEPO-ML model developed in this work (see details in Methods). In Fig. 3, we compare the ML charge models to the physical charge-sharing SQE-MEPO model that was parameterized to replicate REPEAT charges. Parity plots comparing the reference REPEAT charges to the predicted charges are given in Fig. 3a for the SQE-MEPO model, Fig. 3c for the graph attention network (GAT) model, Fig. 3e for the random forest regressor (RFR), Fig. 3f for the gradient boosted decision tree regressor (GBDTR) and Fig. 3g for the gated graph neural network (GGNN) model. The ML models have Pearson *R*^2^’s of 0.97 or greater and the parity plots all look qualitatively similar. On the other hand, the parity plot with the SQE-MEPO method is drastically different with the model having an R^2^ of only 0.75. Similarly, the mean absolute error (MAE) of the predicted charges in the test set for the SQE-MEPO model is 0.186 e, which is at least 4 times higher than the ML models which have MAEs of 0.043 e or less. To further compare the ML models to the physical charge sharing SQE-MEPO model, we examine the distribution of charges. Figure 3b shows a violin plot comparing the charge distributions of REPEAT charges to the distribution of SQE-MEPO charges for the 18 most abundant elements in the test set. The charge distributions of the SQE-MEPO model are strikingly narrow compared to the REPEAT charge distributions which have a much larger variation for all elements shown. The plot clearly highlights the limited capability of the SQE-MEPO method for generating varied charges in different chemical environments. Figure 3d shows the same distribution comparison but for the GAT (MEPO-ML) model where it is fair to say that the GAT charge distributions are near mirror images of the REPEAT charge distributions. Note that the GAT charges after charge neuralization were used to plot Fig. 3d; the same plot using the GAT raw predicted charges can be found in Supplementary Note 1.

Next, we compare the performances of the four ML models against one another, where it is important to note that all models use the same descriptors and a thorough hyperparameter search was performed on each. The MAEs of the charges in the test set range from 0.043 to 0.025 e with the performance of the models having the same ranking as the ML model complexity. More specifically, the least sophisticated decision tree models, the RFR and GBTR have MAEs of 0.043 and 0.037 e, respectively. The RFR model is an ensemble of decision trees whereas the GBTR method improves over the RFR model by iteratively growing more trees to reduce the prediction residuals. The RFR and GBDTR methods only predict the charge based on the descriptors of the atom of interest (including those that describe the atom’s local environment), while the graph neutral networks additionally enable information transfer between chemically bonded atoms via edges encoded in the graphs, thereby giving better predictions. The GGNN gives an MAE of 0.032 e, while the more sophisticated GAT model possesses the best MAE of 0.025 e. It should be noted that previous ML models for predicting the DDEC partial atomic charges reported MAEs of 0.025 e or better. However, it is important to note that the test set used here is completely different than those used in the previous works^25–27^ and therefore the performance metrics are not directly comparable.

**Fig. 4:**
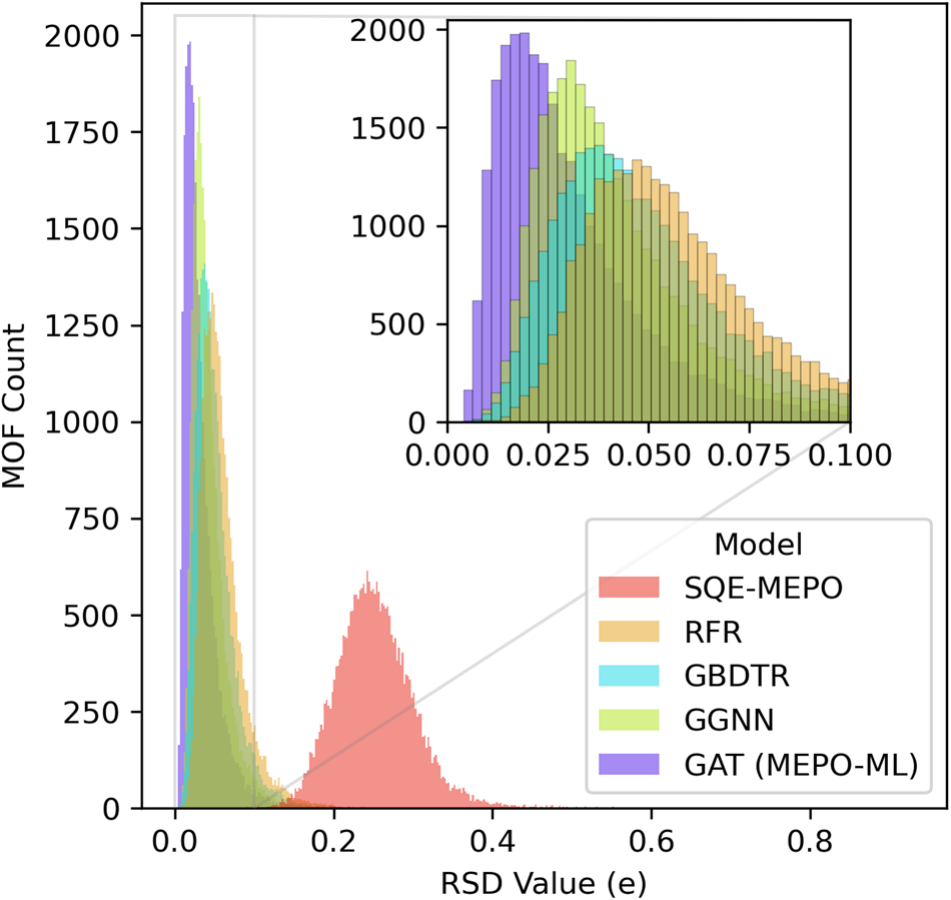
The relative standard deviation (RSD) of each model. Histograms of RSD between the REPEAT charges and predicted charges from the five charge generation models for each MOF in the test set. The inset shows a window to better distinguish the machine learning models.

In addition to evaluating the performance based on each individual atom-based metrics, the prediction errors of each model were also evaluated at the per MOF level using the relative standard deviations (RSD) as proposed by Liu and Luan^36^. The RSD value represents the normalized error of the charge predictions for all atoms in a single MOF, thus, the lower the better. RSD values were computed for all MOFs in our test set for all four ML methods and plotted as histograms in Fig. 4 (with the SQE-MEPO model also given). The trend in the histograms for the ML methods follows the trends as the individual charge based MAEs. Therefore, the GAT model is again shown to be the best performing model for predicting atomic partial charges in MOFs.

### Charge neutrality

Since the ML models are unaware of the total framework charge of the MOFs during training or inference, there is no guarantee that the charges on the atoms will sum to the net charge of the framework, which in this case is zero for all MOFs in the ARC-MOF database. This excess charge must be neutralized, and there are various schemes for achieving this that have been proposed in the literature^23,27,37,38^. The simplest method involves evenly distributing the excess charge across all atomic charges, while more sophisticated methods have been proposed where the excess charge is distributed across the atoms based on a certain error metric. In this work, excess charges are simply evenly distributed across all atoms in a MOF to enforce charge neutrality. The work of Raza et al.^27^ showed that distributing excess charges evenly or based on standard deviations yielded very similar MAE performances for their GGNN model. Moreover, if a model can predict atomic charges very accurately, the amount of excess charge that needs to be distributed would be minimal and therefore the method of the distribution should not be too consequential. To verify this claim, we computed the average charge corrections on the test set for the ML models, listed in Table 1 alongside the MAEs. Note that the “charge corrections” we discuss here are the absolute values of the charges being corrected during the charge neutralization process for each atom. For the RFR and GBDTR models, the average charge corrections are 0.0045 and 0.0026 e, respectively, which is around an order of magnitude smaller than the MAEs of their respective models. This suggested that the performance of the model would not be significantly affected by the charge neuralization scheme. However, this is not the case for GNN models, where GGNN and GAT charges are on average corrected with 0.175 and 0.026 e, respectively. This is likely due to the charge neutrality scheme (Eq. 11) being implemented as part of our GNN models (noted with “in-model charge neutrality” in Table 1). This leads to the GNN models minimizing the loss between the “corrected charges” and the REPEAT charges, instead of the loss between the “raw predicted charges” and the REPEAT charges. When we move the charge neutrality scheme outside the GNN model (i.e. the charges are neutralized after the model inference) and re-train the GNN models, the average charge corrections (noted with “out-of-model charge neutrality” in Table 1) were reduced to 0.0023 e and 0.0024 e for the GGNN and GAT models, respectively; however, as the MAEs of these models are mostly the same, it suggested that whether the charge neutralization scheme is implemented as part of the model does not significantly impact on the overall performance of the GNN models. We note that only “out-of-model charge neutrality” is possible for the decision tree-based models since inference on these models are based on discrete atoms instead of all atoms in the MOF structure. Thus, this investigation suggests that using the even distribution strategy for charge neutrality is reasonable for these ML models and would not introduce any significant errors.

**Table 1:** The average charge corrections on the test set for the ML models

Model	Charge neutrality	Average charge correction (e)	MAE (e)
RFR	Out-of-model	0.0045	0.0430
GBDTR	Out-of-model	0.0026	0.0370
GGNN	In-model	0.1751	0.0320
	Out-of-model	0.0023	0.0321
GAT	In-model	0.0257	0.0247
	Out-of-model	0.0024	0.0248

Charge neutrality: “in-model” means that charge neutrality was enforced during model inference, “out-of-model” means that charge neutrality was enforced after model inference.

### Feature importance for each model

**Fig. 5:**
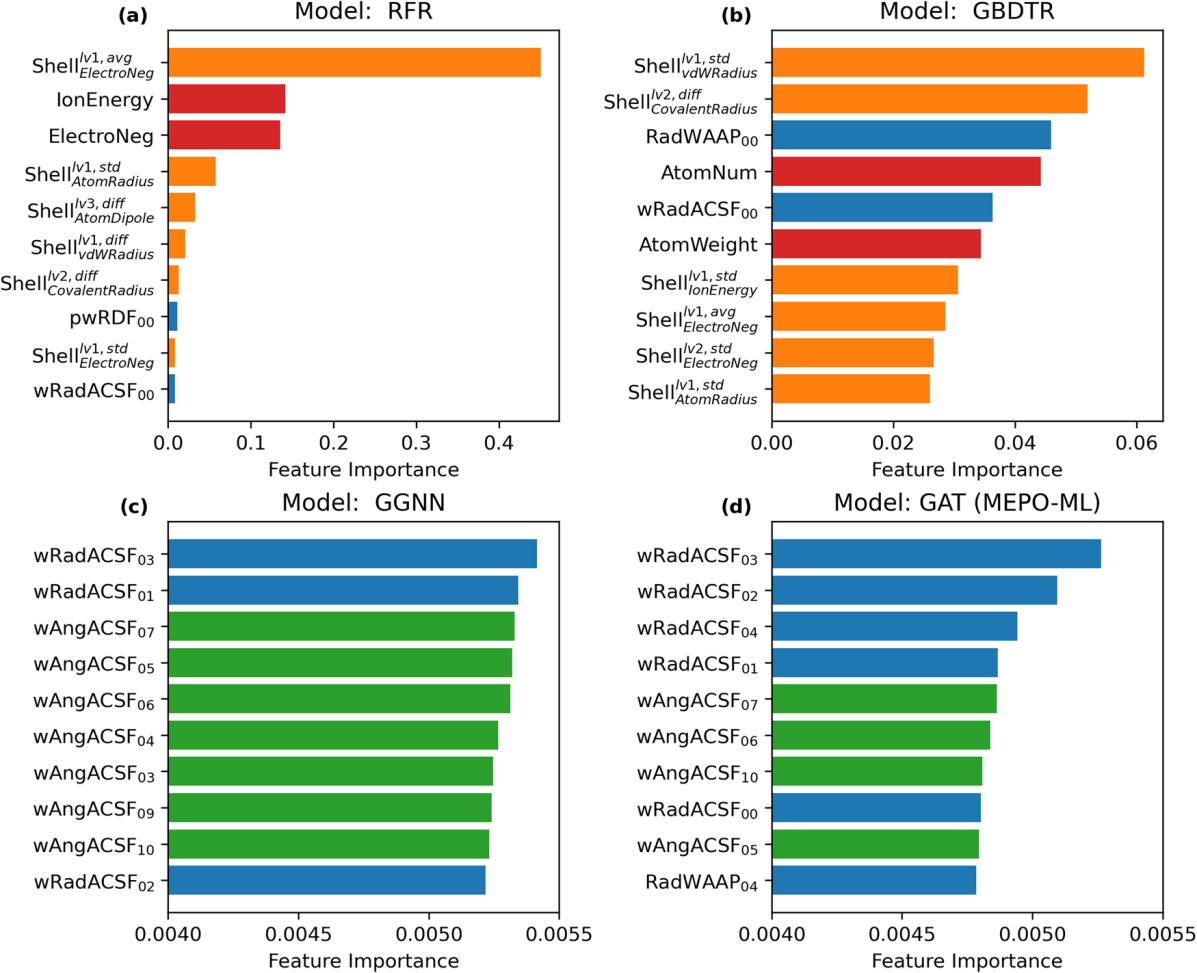
Feature importance of each machine learning model. Bar plots for the top 10 most important feature of (**a**) RFR, **b** GBDTR, **c** GGNN, and (**d**) GAT; the feature importance values are plotted on the *x*-axis with the names of the features plotted on the *y*-axis (see Methods for the full description of the coordination shell descriptor labeling). The bars are colored differently to indicate the type of descriptors: RED = atomic properties, ORANGE = coordination shell descriptors, BLUE = radial function descriptors, GREEN = angular function descriptors.

In this work, 226 descriptors or features are computed for each atom in the database, including 8 atomic properties, 144 shell descriptors and 70 radial function descriptors and 52 angular function descriptors (see details in Methods). One common way for interpreting the models is to compute the feature importance. For the RFR and GBDTR models, the feature importances can be computed from the mean decrease in impurity (or the Gini importance) of the individual decision trees. For explaining the importance of node features in the GNNs, the GNNExplainer^39,40^ provided in the Pytorch Geometric library^41^ is used. It works by determining the variation in prediction accuracy when the certain node features are masked, the larger this variation the more important the masked node features; the feature importance scores are determined for each node, therefore the same feature can have different importance scores on different nodes within the same graph. As such, the reported feature importance is the average of the feature importance scores over all nodes in our test set. Feature importance values are normalized such that they sum to 1 for each model. The top 10 most important features and their feature importance values are plotted in Fig. 5; feature importance values for all features are given in Supplementary Table E2 as a separate spreadsheet. Note that since different methods were used to compute the feature importance, one should not compare them quantitatively, however, a few interesting points can still be made.

The feature importance plots for the RFR and GBDTR models revealed that the coordination shell descriptors (${{Shell}}_{P}^{n,m}$ in Fig. 5) and the atomic properties are the most important descriptors. Both models are not directly aware of the chemical information about the bonded atoms when predicting for an atom of interest, therefore it relies on the covalent shell descriptors to obtain the chemical bonding information. This suggested that the chemical information of bonded atoms is essential for predicting atomic partial charges. Coincidentally, the top 3 most important features in the RFR model match exactly with Kancharlapalli et al.’s work; only 5 descriptors were used for training the RFR model in Kancharlapalli et al.’s work, and the average electronegativity in the first covalent shell, the ionization energy, and the electronegativity were identified as the top 3 most important features^26^. Even though many more features are used in this work, our RFR model still identifies these 3 features as the most important ones.

For the GNN models (i.e., GAT and GGNN), the chemical bonds are encoded as edges in the graphs, and the “message passing” mechanism passes chemical information across the edges via graph convolutions. That is, the essential chemical information of bonded atoms is natively accessible in the GNNs, therefore, the covalent shell descriptors become less important in these models. The ACSF descriptors become dominant in both GNN models, since these descriptors capture 3D through-space environments of an atom, which is not accessible via a chemical graph. However, note that the range of the plots for the GAT and GGNN in Fig. 5 is much smaller than the other plots; in fact, all feature importance are with 0.0040 to 0.0055 for the GNN models, indicating the features are similar in importance for the GNN models.

In the GAT model presented above, bond distances were not encoded as edge features in the graphs for MOFs. One might think that the GNN models could learn the same information from the bond distances as from the 3D coordinate based descriptors (e.g., pwRDFs, ACSFs). However, when training a GAT model from graphs with bond distances as edge features but only atomic identities as node features, the MAE of this model drops significantly to 0.0344 e from 0.0247 e. Additionally, when training a GAT model from graphs that uses both the full descriptor set as node features and bond distances as edge features, only minimal improvement was observed (MAE = 0.0245 e vs 0.0247 e).

On the other hand, with the notion that using too many descriptors in an ML model that are, either not correlated to the properties of interest or that are strongly correlated to one another, may add noise and lower the performance of the models, we examine models trained with reduced feature sets. We first analyzed the covariance matrix of the full feature set of 226 features to examine how the descriptors are correlated; when we removed features that have an absolute covariance coefficient of >0.85 with another feature, we are left with 114 features. When we trained RFR, GBDTR, GGNN, and GAT models with this reduced feature set, the test set MAEs are almost equal to, but slightly worse than the models built with the full feature set was used. One may have expected an improvement using this reduced feature set for the RFR model because the top three most important features (Fig. 5a) already account for 70% of the feature importance for the model. However, the MAE of the RFR model trained with the reduced feature set is 0.0457 e compared to 0.0432 e when trained with the full feature set. For the graph neural network models, GGNN and GAT, the reduced feature set was trimmed down even further to exclude the coordination shell descriptors, since these features should be able to be learned from the message passing within the graph. However, the performance of the models with the reduced feature sets was again slightly worse than with the full feature set. For example, the GAT trained with the reduced feature set is 0.0251 e compared to 0.0247 e when trained with the full feature set.

We also tried a reduced feature set using a principle-component analysis. We found that 99% of the variance was recovered with only 64 principal components. RFR, GBDTR, GGNN, and GAT models were rebuilt with these 64 principle-components as features, but again we found that all models performed slightly worse than their full feature set counterparts. Detailed results for both feature selection analyses are given in Supplementary Note 2.

### Predictions on out-of-distribution geometries

During the curation of the ARC-MOF database, the MOF structures were taken as is from the original experimental and hypothetical MOF databases without additional structural optimizations. Thus, MEPO-ML was trained on a mix of experimental structures from single-crystal X-ray diffraction experiments and computer-generated MOFs taken from various publicly available databases. The hypothetical MOFs were constructed using different assembly methods, in which the geometries were optimized to varying extents using different force field methods. Since MEPO-ML was trained on a variety of equilibrium geometries, one should be able to apply MEPO-ML on any reasonable equilibrium geometry, as is the case with most charge generation models. To study this, we have examined how MEPO-ML performs on “out-of-distribution” geometries where the MOF framework is deformed from the equilibrium via a molecular dynamics (MD) simulation. MD simulations are also a common use case where one may want to apply a rapid charge generation model.

**Fig. 6:**
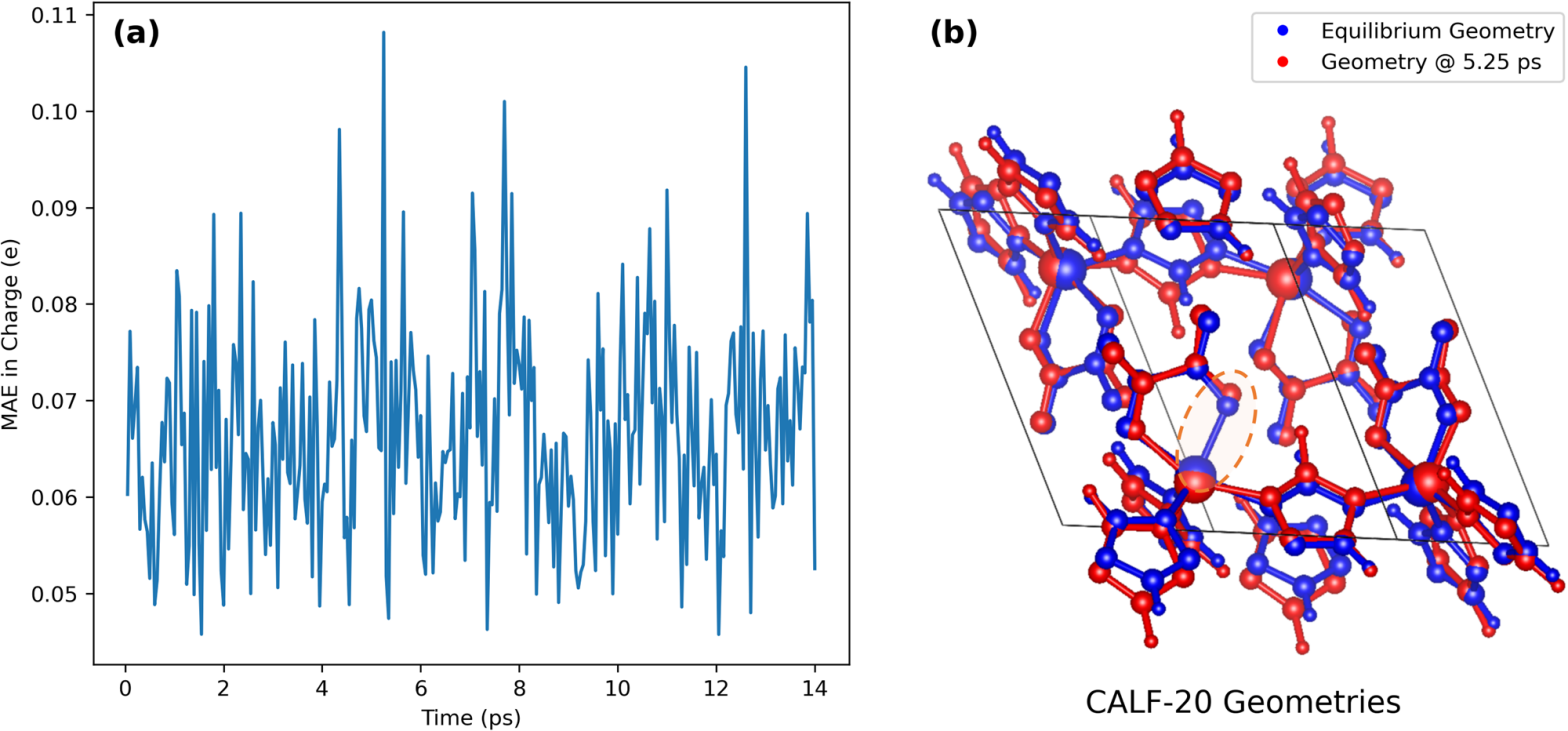
Analyses of the DFT-MD simulation for the MOF CALF-20. **a** Line plots of charge prediction errors (MAE) for CALF-20 over a 14 ps DFT-MD simulation thermostated at 300 K. **b** Comparison of the CALF-20 equilibrium geometry against the structural configuration at 5.25 ps; the orange dashed ellipse highlighted the broken Zn-O bond in the geometry at 5.25 ps.

A constant volume DFT-MD simulation of the MOF CALF-20 was performed using a stochastic Anderson thermostat at 300 K for 14 ps (detailed in Methods). Both REPEAT and MEPO-ML partial atomic charges were computed for configurations that were sampled every 0.05 ps from the MD simulation. Figure 6a presents the charge prediction MAE of each sampled MD configuration during the 14 ps simulation. The average MAE for all 280 MD configurations is 0.067 e. Although this is more than double the MAE of 0.025 e reported for the “equilibrium” test set structures, it is important to realize that CALF-20 is not part of the MEPO-ML training set and the charge prediction MAE of the equilibrium experimental CALF-20 structure is 0.046 e. Thus, MEPO-ML charge predictions are still reasonable for these out-of-distribution geometries. In fact, the prediction MAE with MEPO-ML for these distorted structures are still better than the best QEq charge models when those models are applied to equilibrium structures.

When examining the MD configurations with the large charge prediction errors, it was discovered that one or more of the Zn-O bonds had broken compared to the equilibrium structure. Figure 6b shows the structure with the largest MAE in red compared to the experimental equilibrium structure in blue, where the broken Zn-O bond is highlighted with an orange dashed ellipse. Examination of all 280 configurations revealed that 148 of them possessed broken Zn-O bonds. It is important to realize that geometries with broken bonds were also missing the corresponding edges in their graphs for MEPO-ML charge predictions. This might result in less accurate charge predictions for these geometries since the attention mechanism relies on message passing across chemical bonds, and a “missing edge” would lead to the GAT model interpreting it as a “different MOF”. At the same time, if the Zn-O bond is broken, it might be better to “interpret” it as a different MOF. To examine this, we recomputed the MEPO-ML charges for all 280 configurations but using the same bond table from the equilibrium structure for their graphs (i.e., no broken bonds); this only resulted in a slightly better average MAE of 0.065 e as compared to 0.067 e. This suggests that the MEPO-ML model is able to predict reasonable charges even when the graph structure is changed by geometric distortions.

One question of interest when applying MEPO-ML to an MD simulation is whether recomputing the MEPO-ML charges for each MD configuration gives a better charge prediction when compared to simply using the same MEPO-ML charges computed from the equilibrium geometry. If one fixes the charges to the MEPO-ML charges from the equilibrium structure for each step of the MD simulation, the average MAE compared to DFT-derived charges is 0.080 e. This compares to the average MAE of 0.067 if MEPO-ML charges are recomputed for every step. This result further suggests that MEPO-ML can provide reasonable charge predictions for out-of-distribution geometries.

### Effect of data quantity for training

**Fig. 7:**
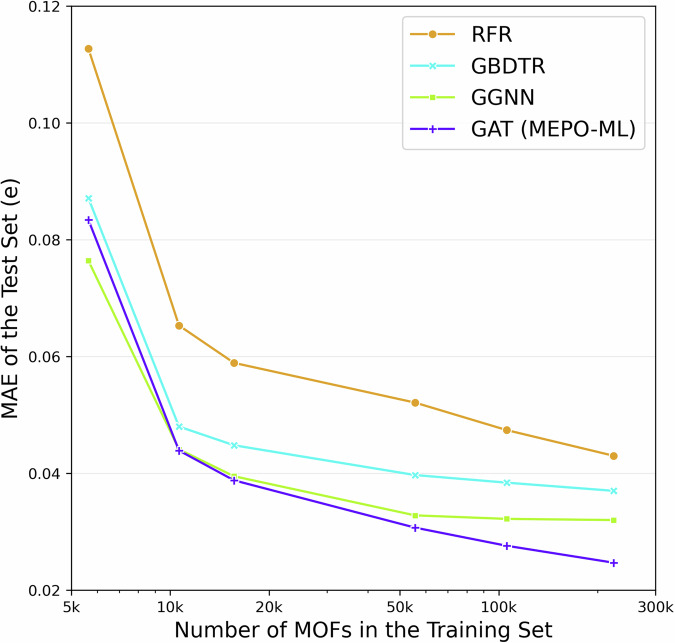
Performance analysis of varying training set sizes. Line plots of the mean absolute error of the partial atomic charges predicted as a function of the training set size with different machine learning models, where the x-axis is given in logarithmic scale.

The three previously reported ML models for predicting partial atomic charge predictions in MOFs used datasets of less than 3000 MOFs from the CoRE databases^25–27^. In this work, the total training set consisted of over 200,000 MOFs. To examine the difference in performance when using training sets of smaller sizes with the various models, 5 smaller training sets were extracted from the total training set used in this work. The first training set is the subset of 5565 CoRE MOFs in the total training set. This set is roughly equivalent to the datasets used to train previous ML models. Starting from the second dataset, additional MOF were sampled from the total training set and added to the first set to create the new datasets of increasing size. Rather than randomly choosing additional MOFs, we wanted to create diverse training sets. To do this, we used revised autocorrelation function (RAC) descriptors for MOFs developed by Moosavi et al.^42^ to characterize the metal nodes and organic linker (20 RAC descriptors for each) of the MOFs in the total training set. We then used furthest point sampling of MOFs based on their RAC descriptors to add an additional 5000/10,000/50,000/100,000 MOFs to the first dataset to create the 2^nd^/3^rd^/4^th^/5^th^ training sets, respectively. More details about the RAC descriptors and the sampling are discussed in Supplementary Note 3. We then trained the RFR, GBDTR, GGNN, and GAT models using these 5 training sets and tested them on the original test set of 27,996 MOFs. Plots of the charge MAEs of the four models trained on the datasets of increasing size are given in Fig. 7, where it is important to note that the same test set was used in all cases. The general trend for all models follows the intuition that more training data results in better prediction performance. Furthermore, notice that the two GNN models always outperform the RFR and GBDTR models with the same training set size, suggesting algorithmic advantages of the GNNs for both small and large training sets. Of the two GNNs, it is interesting to note that the GGNN outperforms the GAT with the smallest training set. However, the GAT model continues to improve with increasing training set size whereas the performance of the GGNN appears to level off after a training set size of ~50 K. The data point for the GAT model with the largest sized training data corresponds to the MEPO-ML model. The trend line for the GAT model given in Fig. 7 suggests that the MEPO-ML model can be further improved with more training data.

### Analysis of anomalous MEPO-ML charges

Here we investigate cases where the MEPO-ML charges deviate significantly from the REPEAT charges in the test set. We first examined MOFs in the test set which contained atoms where the MEPO-ML charge differed from the REPEAT charge by more than 1.0 e; there were 45 such MOFs. For 24 of these MOFs, we found that there were errors in processing the REPEAT charges that were fit from the DFT calculations. With more than 270 K MOFs in the dataset, it is perhaps not surprising that the high throughput workflow used to process the charges observed some such errors. When the REPEAT charges are corrected, the MAE between the MEPO-ML and REPEAT charges in these 24 MOFs drops to 0.020 e from the 0.271 e observed prior to correction.

For the remainder of the 21 MOFs with charges differing by more than 1.0 e, we examined the structures in detail. 10 of the 21 structures contained obvious structural errors such as missing protons, disordered atoms, or missing counterions—the latter of which means that the DFT calculation was performed on a neutral framework, whereas the true framework has a net charge. The remaining 11 structures possessed unusual geometric features such as non-linear alkyne moieties, distorted aromatic rings, or potentially undercoordinated metal atoms. Five of the MOFs with unusual geometries originated from the Boyd-Woo hypothetical MOF database^7^, in which case the distortions were likely an artifact of the geometry optimization that was performed at the force field level. A detailed account of the structural errors or unusual structural features found in the 21 MOFs is provided in Supplementary Note 4.

Next, we examine partial charges for elements that are absent in the training set – specifically Pu, Rh and Te (see Fig. 1b). In each case, the test set contains only a single experimental MOF with the rare element and each MOF contains only a single symmetry equivalent site for that element. Table 2 gives the REPEAT and MEPO-ML predicted charges for the element in the test set, along with the RSD value for the whole MOF. The worst performing charge is for the Te atom in the experimental MOF DB12-OROJEM_freeONLY, where the REPEAT charge is 1.816 e while MEPO-ML predicted it as 0.972 e – a difference of 0.844 e. We note that the Te atom is part of the [TeMo_6_O_24_]^6−^ polyoxoanion and is coordinated to 6 highly electronegative oxygen atoms, which likely accounts for the large, positive REPEAT charge. Additionally, we don’t expect too many similar polyoxoanion moieties in ARC-MOF since they are not contained in any SBU libraries (that we are aware of) for building hypothetical MOFs, and ARC-MOF is composed of ~97% hypothetical MOFs. Thus, the model may not be able to predict charges well for atoms in such environments.

**Table 2:** Comparison of REPEAT and MEPO-ML charges for elements not in the training set

MOF label	Element	*q*^REPEAT^	*q*^MEPO-ML^	|Δ*q*|	MOF RSD
DB12-TAGCIP_clean	Pu	2.174	2.030	0.144	0.186
DB12-TERFUT_freeONLY	Rh	0.416	−0.053	0.469	0.157
DB12-OROJEM_freeONLY	Te	1.816	0.972	0.844	0.171

Note: In each case there is only a single MOF in the test set with the element in a single symmetry equivalent site. Charges given in units of electron charge (*e*). “$\Delta{q}$” is the difference between the two partial atomic charges and RSD is relative standard deviation of the MOF.

For the Rh-containing MOF DB12-TERFUT_freeONLY, the REPEAT charge was 0.416 e while the MEPO-ML model predicted to be −0.053 e. Here we note that the Rh center in DB12-TERFUT_freeONLY is highly under-coordinated with a rare coordination number of 2 and non-linear geometry. Finally, for Pu, there is a 0.14 e difference in the REPEAT and MEPO-ML charges. Although this difference is significantly better than for Rh and Te cases, it is still significantly higher than the MAD of 0.025 e observed for all atoms in the test set. We note that for all three MOFs given in Table 2 the RSD, which quantifies the charge deviations for the whole MOF, are all >0.15 e, comparing to an average RSD of 0.03 e for all MOFs in the test set. Thus, as one might expect, MEPO-ML does not perform well for MOFs containing elements not seen in the training set.

### Gas adsorption simulations

**Fig. 8:**
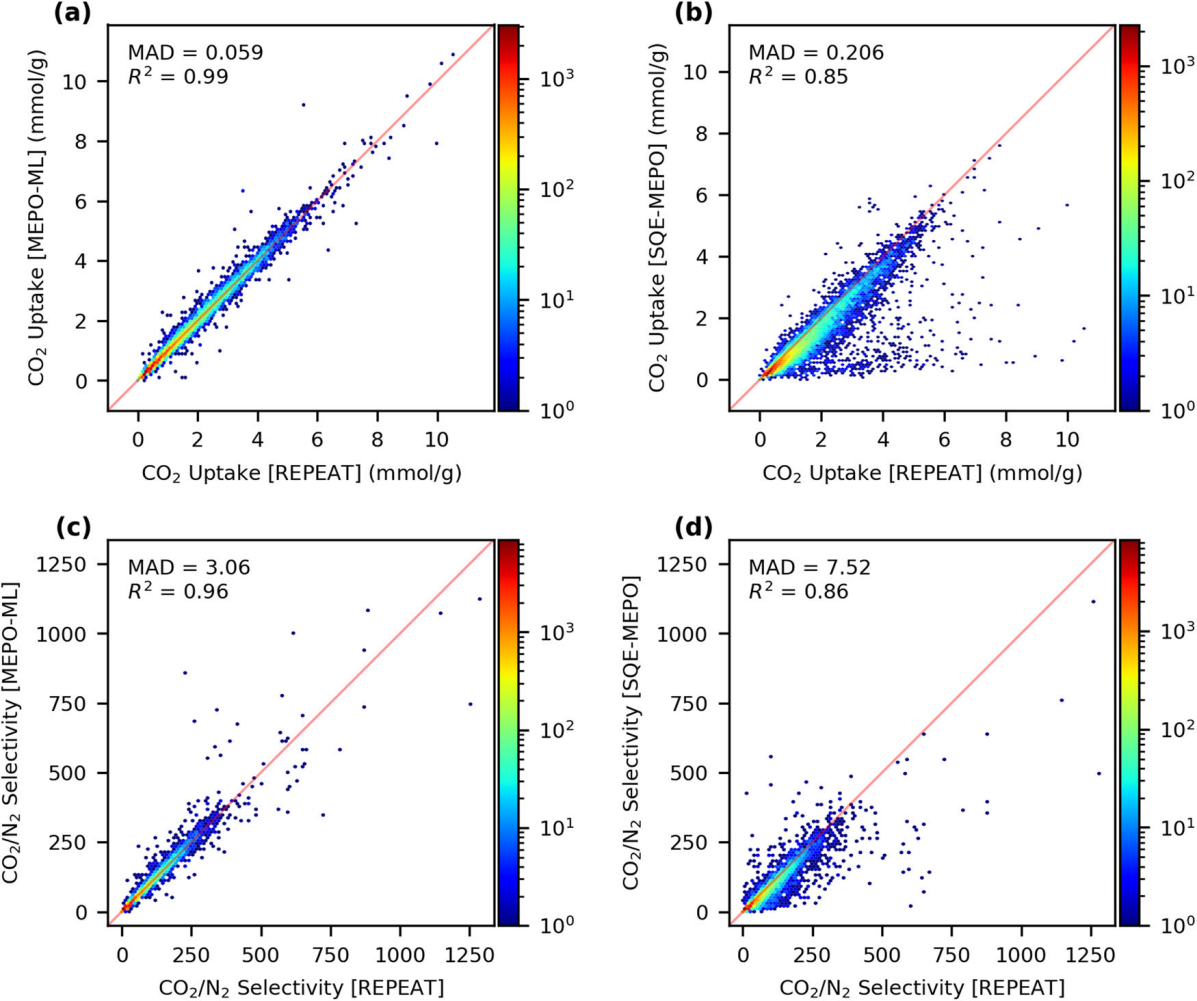
GCMC simulation at post-combustion conditions. Simulation results of the test set (27,996 MOFs) at 1 bar, 298 K with a CO_2_/N_2_ ratio of 15:85. **Top**: Parity plots of CO_2_ uptake computed with (**a**) MEPO-ML charges and (**b**) SQE-MEPO charges compared to the benchmark results computed with REPEAT charges. **Bottom**: Parity plots of the CO_2_/N_2_ selectivity computed with (**c**) MEPO-ML charges and (**d**) SQE-MEPO charges compared to the benchmark results computed with REPEAT charges.

To examine the effects of different charge assignment schemes in evaluating physical properties, the adsorption properties of various gases were computed with atomistic GCMC simulations on the test set of 27,996 MOFs. First, we evaluate the CO_2_ adsorption properties at post-combustion carbon capture conditions with a 15:85 CO_2_/N_2_ binary gas-phase mixture at 1 bar and 298 K. Figure 8 gives parity plots of CO_2_ uptakes and the CO_2_/N_2_ selectivities for MOFs in our test set using MEPO-ML charges and SQE-MEPO charges compared to the results using DFT-derived REPEAT charges. The MEPO-ML charges perform exceptionally well for the CO_2_ uptakes where the mean absolute deviation (MAD) compared to the REPEAT model is only 0.059 mmol/g. This compares to the MAD of 0.206 mmol/g with the SQE-MEPO model, which is over three times larger. The CO_2_/N_2_ selectivity shows a similar performance gap between the two models, with the MEPO-ML model giving a MAD of 3.06, which is 2.5 times smaller than the MAD of 7.52 for the SQE-MEPO model. We note that there are some expected deviations between the results using REPEAT charges and the other charge models due to the finite sampling and stochastic nature of the GCMC simulations, particularly when the uptake is very low.

**Fig. 9:**
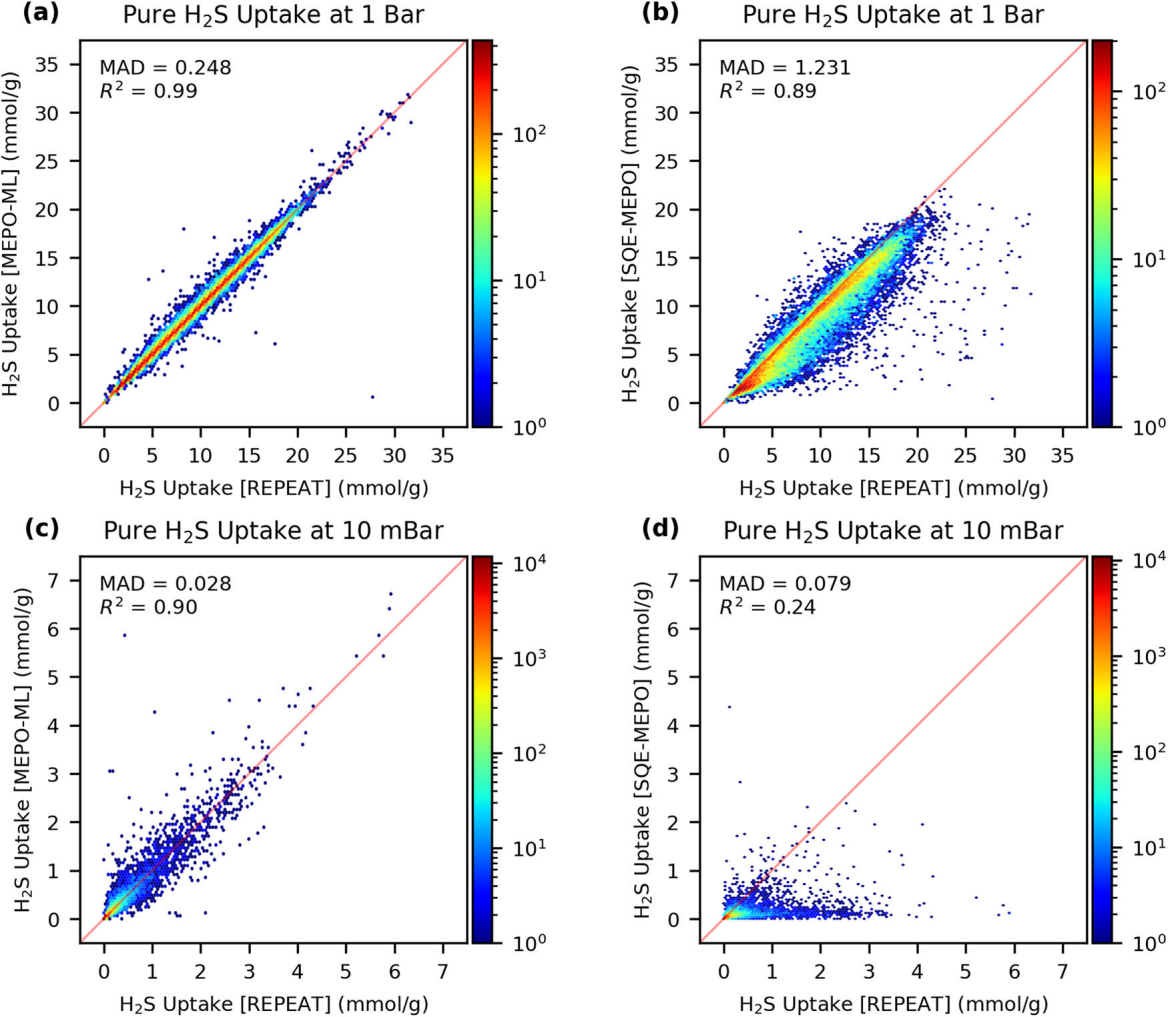
GCMC simulation for pure H_2_S. Simulation results of the test set (27,996 MOFs) for pure H_2_S adsorption at 298 K. **Top:** Parity plots of the H_2_S uptake at 1 bar computed with (**a**) MEPO-ML charges and (**b**) SQE-MEPO charges against the benchmark results with REPEAT charges. **Bottom:** Parity plots of the H_2_S uptake at 10 mbar computed with (**c**) MEPO-ML charges and (**d**) SQE-MEPO charges against the benchmark results with REPEAT charges.

To further demonstrate the importance of accurate charges in gas adsorption simulations, we examine the adsorption properties with a polar gas at both low and high pressure. For this we examined the H_2_S uptake at 1 bar and 10 mbar, where the latter pressure is relevant for biogas applications^43^. Fig. 9 shows parity plots of the H_2_S uptake capacity computed using MEPO-ML and SQE-MEPO charges compared to those computed with REPEAT charges. At 1 bar, the MEPO-ML model has an MAD in the H_2_S uptake of 0.25 mmol/g which is almost five times better than the MAD of 1.23 mmol/g of the SQE-MEPO model. At 10 mbar, the MAD is 0.028 mmol/g for the MEPO-ML model and 0.079 mmol/g for the SQE-MEPO model, which is only about 3 times better. While the performance gap seems to be reduced at low pressure when examining the MADs, it is important to note that the uptakes are much lower at 10 mbar such that the absolute errors are smaller than at higher pressure. Examination of the parity plot of the SQE-MEPO model, given in Fig. 9d, shows the SQE-MEPO model systematically under-predicts the uptake. This is reflected in the Pearson R^2^ of the SQE-MEPO model, which is rather poor at 0.24. In comparison, the R^2^ is still quite high at 0.90 for the MEPO-ML model. For predicting the uptake of a polar gas where electrostatic interactions are expected to play an important role, the more accurate MEPO-ML model significantly outperforms the SQE-MEPO model at both high and low pressure.

Rapid charge generation schemes are often used for high throughput screening of materials, where the recovery of top performers is important. As such, we examined how well the MEPO-ML and SQE-MEPO models recover the top 300 MOFs (c.a. top 1%) from our test set of 27,996 MOF structures screened with the DFT-derived REPEAT charges. Table 3 provides these results for the CO_2_ uptake and CO_2_/N_2_ selectivity at post-combustion conditions, as well as H_2_S uptake at 1 bar and 10 mbar. The results indicate that the MEPO-ML method has a high recovery rate and significantly outperforms the SQE-MEPO method in all scenarios. While the SQE-MEPO screenings perform respectably for post-computation CO_2_ capture, is only able to recover less than half of the higher performing materials for H_2_S adsorption at 1 bar and less than 20% at 10 mbar. Presumably the performance gap between the models is greater for H_2_S uptake because the more polar nature of the guest makes the results more sensitive to the partial atomic charges used.

**Table 3:** Number of the top 300 performers in terms of uptake or selectivity that the MEPO-ML and SQE-MEPO charge models recover compared to using the reference DFT-derived REPEAT charges for different gas adsorption properties

Property	MEPO-ML	SQE-MEPO
Post-combustion CO_2_ uptake	273 (91%)	190 (63%)
Post-combustion CO_2_/N_2_ selectivity	260 (87%)	208 (69%)
H_2_S uptake at 1 bar	271 (90%)	143 (48%)
H_2_S uptake at 10 mbar	238 (79%)	57 (19%)

### Deployment of MEPO-ML

**Fig. 10:**
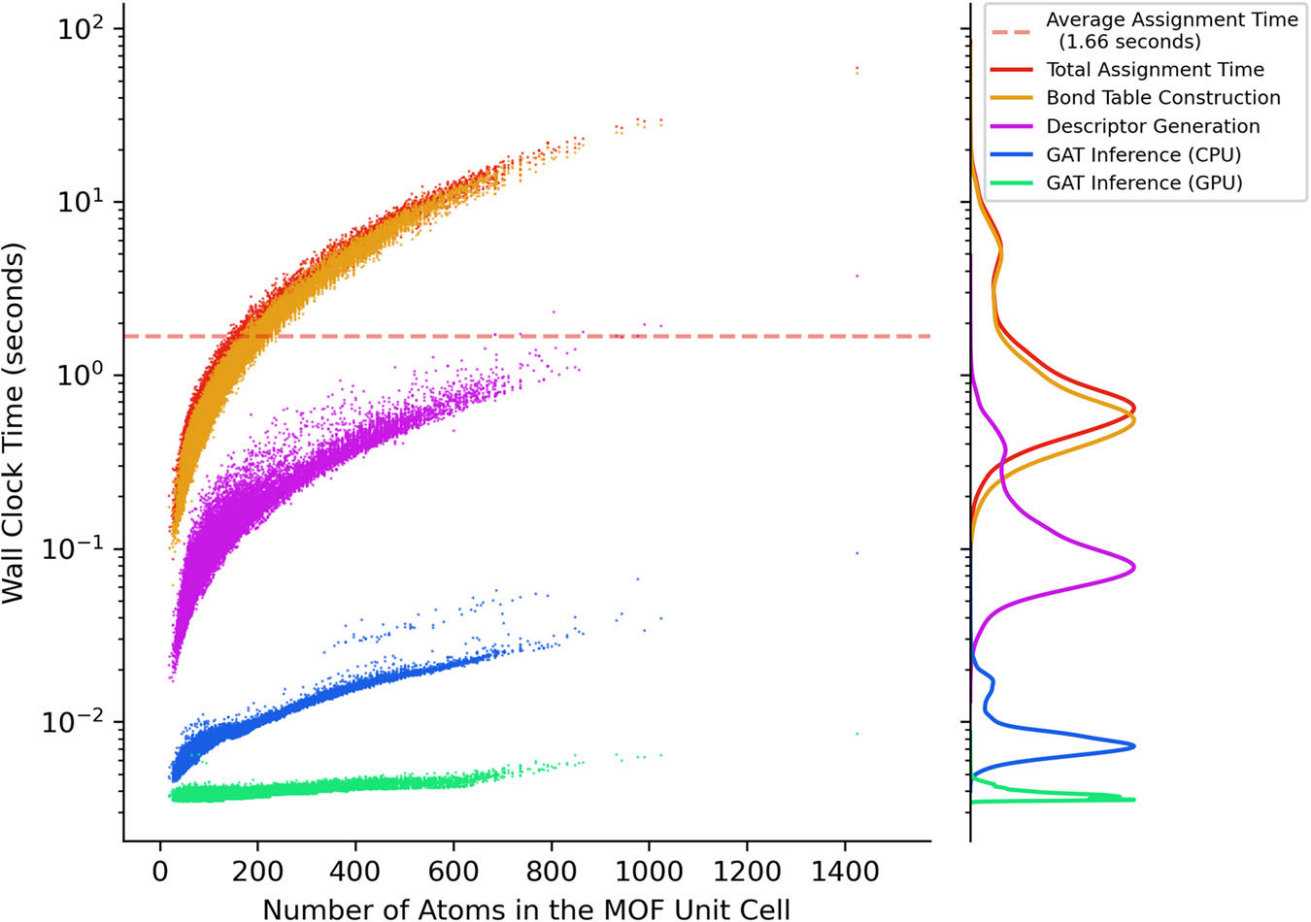
Charge generation speed benchmarks. A scatter plot for wall-clock times to compute MEPO-ML charges against the number atoms in the unit cell for the entire test set MOFs (27,996 MOFs). Note that the *y*-axis is in logarithmic scale. The kernel density estimate plots for each component of the benchmark time breakdowns are displayed on the side. The pale dashed red line indicates the average total charge assignment time for the entire test set.

A charge assignment code for using our pre-trained MEPO-ML model is written in Python and provided on our GitHub repository for public usage. The charge assignment code takes a MOF structure in the CIF format as input and writes a new CIF file with predicted charges embedded in the file. The code can generate charges rapidly even with a single CPU core. Figure 10 shows the wall-clock times per MOF on a consumer grade desktop computer (with an AMD 5900X CPU and Nvidia RTX 3070 GPU) for generating the charges on the entire test set MOFs (~28 K structures); only one CPU core is used for this benchmark. Aside from the total charge assignment time, the benchmark also records times required to (a) generate the bond connection table required to construct the graph, (b) compute the descriptors, and (c) compute the charges from the GAT model inference. The bond table construction is by far the most time-consuming part of the calculation (orange contents in Fig. 10), where we used the Isayev nearest neighbor algorithm^44^ implemented in the Pymatgen library^45^. For MOFs with a very large unit cell (over 1400 atoms), the bond table construction alone can take over a minute. However, it should be noted that over 80% of the MOFs in the ARC-MOF database have less than 200 atoms in the unit cell. Therefore, in most cases, our code can generate partial atomic charge embedded CIF files within 2 seconds with a single CPU core. For the final charge prediction step, the GAT model inference times using both CPU (blue) and GPU (green) are plotted in Fig. 10. While using the GPU gives much faster charge prediction times, it only takes less than 0.4 s when using the CPU. We also tested our charge assignment code in a parallel workflow for simultaneous charge assignment on multiple MOFs across all 12 cores (24 threads) on the AMD 5900X CPU in our benchmark computer. Charge assignments on all entries of our test set (27,996 MOFs) finished within 3 h—this is less than 0.4 seconds per MOF on average. This suggest our charge assignment would also be suitable for high-throughput tasks on systems with high CPU core counts.

## Discussion

In this work, we presented the MEPO-ML model based on the graph attention network (GAT) ML architecture for generating fast and accurate partial atomic charges for MOFs. With a GAT model, the MOF structure is converted into a graph with atoms as nodes and bonds as edges, where descriptors with chemical environment information are encoded as node and edge features. The MEPO-ML model was trained and tested with the ARC-MOF database containing ~279 K MOF structures and over 40 million DFT-derived REPEAT partial atomic charges. Previous ML models used to predict partial atomic charges in MOFs used datasets containing less than 3 K MOFs that likely possess large structural error rates^34^.

We compare our MEPO-ML model against other ML architectures used in the literature for predicting partial atomic charges in MOFs, namely a random forest regressor (RFR), a gradient boosted decision tree regressor (GBDTR) and a gated graph neural network (GGNN). For partial atomic charge predictions, the GAT-based MEPO-ML model outperformed these 3 other ML models, with an MAE of 0.025 e and an *R*^2^ of 0.99 against the DFT-derived charges, using the same training set and descriptor sets. Additionally, results from training the models with various training set sizes suggests that accuracy of the MEPO-ML model can be further improved with even more training data. We also compare the MEPO-ML model to the empirical SQE-MEPO model for both charge predictions as well as gas adsorption performance via GCMC simulations. When evaluating on the same test set, the SQE-MEPO method has an MAE in the predicted charges of 0.186 e, which is almost an order of magnitude higher than MEPO-ML’s MAE. When evaluating gas-adsorption properties, we find that the results using MEPO-ML charges are in much better agreement with those determined with the DFT-derived charges as compared to those using SQE charges. For example, for evaluating H_2_S uptakes at 10 mbar, the Pearson R^2^ for MEPO-ML was 0.90 and whereas for SQE-MEPO it was only 0.24. Furthermore, MEPO-ML was able to recover 238 of the top 300 performing MOFs from the H_2_S screening at 10 mbar with the reference REPEAT charges whereas SQE-MEPO was only able to recover 57 of them.

With such drastic improvements in both the predicted charges and computed adsorption properties using the charges, we recommend that MEPO-ML charges replace the use of empirical methods charge sharing models, such as QEq or SQE, in high-throughput virtual screening workflows.

## Methods

### Dataset preparation

The ARC-MOF database^9^ contains 279,632 MOFs with over 40 million DFT-derived REPEAT partial atomic charges labeled for every atom in the unit cell of each MOF. We note that the structures in ARC-MOF were screened for structural errors based on our in-house code that computes the metal oxidation states of all metal atoms for the structure as given. If any metal oxidation state in a structure is found to be impossible, unknown or extremely rare, the structure is flagged as having an error. The method was validated on over 16 K structures from the CoRE 2019 database that were all manually inspected for structural errors by comparing the structure to that described in the original publication. If a structure was flagged as having an error, it was found to contain an error 95% of the time based on our manual validation. Like all electrostatic potential fitting charge methods, the REPEAT method can be prone to the so-called buried atom problem, which can result in “unphysically” large charges. MOF structures with unusually large charges that may result from problems with the electronic structure, processing errors, or buried atoms were removed during the curation of ARC-MOF. Further details of the curation process of the ARC-MOF database were discussed in the original publication^9^.

The database was split into training, development and test sets in an 80:10:10 ratio. As discuss in the Results, since the population of elements in the ARC-MOF database varies by several orders of magnitude (see Fig. 1a), random splitting is likely to arbitrarily bias or exclude certain elements from the splits. Therefore, MOF structures were split such that the fraction of each element observed in each split roughly maintains the 80:10:10 ratio. To achieve this, the atom counts of each element contained in each MOF was determined. Then 10 bins were created to replicate the 80:10:10 split where 8 bins belonged to the training set and 1 bin each belonged to the development and test sets. Starting from the least common element, all MOFs containing this element were randomized and put into each bin sequentially as to populate each bin evenly with MOFs. Then the procedure was repeated for the next least common element until all 279 K MOFs were placed into one of the 10 bins. All MOFs in the first 8 bins were combined as the training set, while the MOFs in the last two bins made up the development and test sets, respectively. The percentages of the resulting splits are illustrated in Fig. 1b–d.

### Graph construction

MOF structures stored in the crystallographic information file (CIF) format need to be converted to graphs as the input for a graph neural network (GNN). For MOFs, nodes in a graph are simply the atoms in the unit cell of the MOF, while the edges connecting the nodes represent the chemical bonds between the atoms. Chemical bonds, including those across the periodic boundary, are found using the Isayev nearest neighbor algorithm^44^ implemented in the Pymatgen library^45^, where two atoms are considered bonded if (i) they share a Voronoi facet and (ii) the bond distance is less than the sum of the Cordero covalent radii, plus 0.5 Å. By default, the algorithm uses a radial tolerance of 0.25 Å for the sum of covalent radii, however, we noticed that this default tolerance often fails to capture bonds between metal atoms and carboxylates, which is a common bond-type in MOFs. In some cases, this results in isolated nodes or disconnected graph components when encoding the graphs for MOFs. Therefore, we opted to increase the tolerance to 0.5 Å in this work. After constructing all graphs with the 0.5 Å tolerance, for all MOFs in our dataset, no isolated nodes were found using PyTorch Geometric’s “*has_isolated_nodes*” function^41^ and only one graph component for each MOF was found using NetworkX’s “*connected_components*” function^46^, i.e., all nodes are connected as a single graph for all MOFs in our dataset.

### Atomic descriptors

The chemical environments of an atom in a chemical system can be encoded into numeric vectors with physical properties of atoms and values calculated from descriptor functions. For ML models that take tabulated data as inputs, such as decision tree models, these atomic descriptors are directly used as the inputs. For graph neural networks, atomic descriptors are provided as node features in the chemical graph. In this work, eight atomic properties are chosen to describe each atom’s elemental identity: the atomic number, the atomic weight, the van der Waals radius, the first ionization energy, the Cerdero covalent radius, the Rahm atomic radius^47^, the static dipole polarizability^48^ as well as the Ghosh electronegativity^49^. These specific properties are selected because they have entries for all 74 elements in the ARC-MOF database and were readily available from the Mendeleev Python library^50^. Since the graph representation of a MOF only captures the bonding relationships among the atoms, descriptors that capture an atom’s radial and angular local environments are also added to the node feature vector. Four types of descriptors are used in this work: the atomic property weighted distribution functions, the weighted average atomic properties, the weighted atom-centered symmetry functions and the coordination shell descriptors.

The atomic property weighted radial distribution functions (*pwRDFs*) were developed by Woo and coworkers to describe the chemical and geometric features of an entire MOF structure for gas adsorption property predictions^51^. For this work, the formulation of *pwRDF* is modified to capture the local environment of an atom, as given in Eq. 1, where $i$ indicates the atom whose local environment is being captured, $j$ is a neighbor atom to $i$ within a certain radial cut-off (${R}_{{cut}}$) with ${r}_{{ij}}$ being the distance between the two atom, $N$ is the total number of neighbors within ${R}_{{cut}}$, $P$ is an atomic property (such as electronegativity), and $\alpha$ is a Gaussian smoothing parameter. The term inside the summation computes a radial feature for each neighbor $j$ based on the distance of interest $R$; as such, the descriptor ${{pwRDF}}_{i}\left(R\right)$ encodes the sum of atomic radial features of all neighbors at distance $R$ from the atom $i$.

1 $${{pwRDF}}_{i}\left(R\right)=\mathop{\sum }\limits_{j\ne i}^{N}{P}_{j}\cdot {e}^{-\alpha {\left({r}_{{ij}}-R\right)}^{2}}$$

Moreover, we extend the *pwRDF* approach to capture the local angular environment of an atom with the atomic property weighted angular distribution function, *pwADF*, given in Eq. 2. The summations enumerate over neighbor atoms $j$ and $k$ that are within a cutoff (${R}_{{cut}}$) of the central atom $i$, that form the angle ${\theta }_{{jik}}$. ${P}_{j}$ and ${P}_{k}$ are atomic properties of atoms $j$ and $k$, respectively, while $\beta$ is a Gaussian smoothing parameter. It should also be noted that the set of neighbors $N$ can be different for *pwRDF* and *pwADF* if a different radial cutoff (${R}_{{cut}}$) is chosen for the two descriptor functions. The descriptor ${{pwADF}}_{i}\left(\Theta \right)$ encodes the sum of the angular features subject to the angle of interest $\varTheta$ around atom $i$.

2 $${{pwADF}}_{i}\left(\varTheta \right)\,=\mathop{\sum }\limits_{j\ne i}^{N}\mathop{\sum }\limits_{k\ne i,j}^{N}{P}_{j}\cdot {P}_{k}\cdot {e}^{-\beta {\left({\theta }_{{jik}}-\varTheta \right)}^{2}}$$

Next, we explore a variation of the *pwRDF* and *pwADF*, where a weighted average of the neighbors’ atomic properties based on the distance and angle of interest, $R$ and $\varTheta$, are calculated. We call these the radial and angular weighted average atomic properties (*RWAAP* and *AWAAP*) and are given in Eqs. 3 and 4. These descriptor functions take the form of the weighted average, where the atomic properties of the neighbors (i.e., ${P}_{j}$ and ${P}_{k}$) are weighted by the radial or the angular features (the Gaussian term).

3 $${{RWAAP}}_{i}\left(R\right)=\frac{\sum_{j\ne i}^{N}{P}_{j}\cdot {e}^{-\alpha {\left({r}_{{ij}}-R\right)}^{2}}}{\sum_{j\ne i}^{N}{e}^{-\alpha {\left({r}_{{ij}}-R\right)}^{2}}}$$

4 $${{AWAAP}}_{i}\left(\varTheta \right)=\frac{\sum_{j\ne i}^{N}\sum_{k\ne i,j}^{N}{P}_{j}\cdot {P}_{k}\cdot {e}^{-\beta {\left({\theta }_{{jik}}-\varTheta \right)}^{2}}}{\sum_{j\ne i}^{N}\sum_{k\ne i,j}^{N}{e}^{-\beta {\left({\theta }_{{jik}}-\varTheta \right)}^{2}}}$$

The third set of descriptors for the node feature vector are the atom-centered symmetry functions (ACSFs) which were first developed by Behler in 2011 for potential-energy surface predictions^31^. Gastegger et al.^52^ adopted the weighted version to overcome the undesirable scaling of the number of ACSF descriptors as the number of chemical elements in the system increased. The weighted radial and angular ACSFs used in this work will be referred as *wRACSFs* and *wAACSFs*, respectively. The exact forms of the functions used here are given in Eqs. 5–7. The formulation of the *wRACSF* (Eq. 5) is similar to *pwRDF* (Eq. 1) but with the additional term of the cut-off function (${f}_{{cut}}$). This cut-off function (Eq. 7) gives a larger value as ${r}_{{ij}}$ approaches 0, therefore emphasizing short-range radial features. Note that the Gaussian smoothing parameter ($\gamma$) and the distance of interest ($\mu$) in the *wRACSF* have different symbols to differentiate them from similar parameters defined in previously described descriptors. The *wAACSF* (Eq. 6) captures the angular features with the angular term ($1+\lambda \cdot {\theta }_{{jik}}$), where ${\theta }_{{jik}}$ is the angle spanned by the atom of interest $i$ and two neighbors $j$ and $k$. The phase parameter $\lambda =\pm 1$ is the phase of the angular term on a polar plot. Gastegger et al.^52^ suggested to generate different angular descriptors using different smoothing parameters ($\eta$ in Eq. 6) of the Gaussian term, which is what we adopted here. Compared to the original form of *wAACSF*, our formulation omits the $\zeta$ parameter (an exponent term) and the $\mu$ parameter (a Gaussian shifting term) that was in the original formulation since they are set to $\zeta =1$ and $\mu =0$; more details about the parameters in the weighted ACSFs were discussed in the work by Gastegger et al.^52^

5 $${{wRACSF}}_{i}\left(\mu \right)=\mathop{\sum }\limits_{j\ne i}^{N}{P}_{j}\cdot {e}^{-\gamma {\left({r}_{{ij}}-\mu \right)}^{2}}\cdot {f}_{{cut}}\left({r}_{{ij}}\right)$$

6 $${wAACS}{F}_{i}\left(\lambda ,\eta \right)=\mathop{\sum }\limits_{j\ne i}^{N}\mathop{\sum }\limits_{k\ne i,j}^{N}{P}_{j}\cdot {P}_{k}\cdot \left(1+\lambda \cdot {\theta }_{{jik}}\right)\cdot {e}^{-\eta {\left({r}_{{ij}}+{r}_{{ik}}+{r}_{{jk}}\right)}^{2}}\cdot {f}_{{ij}}\cdot {f}_{{ik}}\cdot {f}_{{jk}}$$

7 $${f}_{{cut}}\left({r}_{{ij}}\right)={f}_{{ij}}=\frac{1}{2}\left[\cos \left(\frac{\pi {r}_{{ij}}}{{R}_{{cut}}}\right)\right]$$

Lastly, descriptors based on the average atomic properties of coordination shells of an atom in a system were used to train the RFR model in the work from Kancharlapalli et al.^26^. An illustrative figure of coordination shells is provided in Supplementary Note 5. For this work, we compute three different types of coordination shell descriptors (Eqs. 8–10), where the superscript $n$ indicates the level of coordination shell, the second superscript (“avg”, “diff” or “std”) indicates the type of descriptor being calculated (average, difference, or standard deviation, respectively), the subscript $P$ indicates an atomic property, $i$ indicates the atom of interest, $N$ is the number of atoms in the coordination shell, $j$ indexes the atom in the coordination shell, and $\bar{{P}_{j}}$ is the average atomic property in the coordination shell. The number of coordination shell descriptors depends on the number of levels of the covalent shells ($n$) and the number of atomic properties ($P$).

8 $${{Shell}}_{P}^{n,{avg}}\left(i\right)=\mathop{\sum }\limits_{j}^{N}\frac{{P}_{j}}{N}$$

9 $${{Shell}}_{P}^{n,{diff}}\left(i\right)=\mathop{\sum }\limits_{j}^{N}\frac{\left|{P}_{i}-{P}_{j}\right|}{N}$$

10 $${{Shell}}_{P}^{n,{std}}\left(i\right)=\sqrt{\mathop{\sum }\limits_{j}^{N}\frac{{\left({P}_{j}-\bar{{P}_{j}}\right)}^{2}}{N}}$$

The atomic properties and the abovementioned descriptors of each atom are concatenated to yield the node feature vector for each atom. Due to the large numerical variance in these features, all node features are standard scaled based on features in the training set. Codes for all descriptor computations are implemented in our own Python codes; the exact parameters used for the descriptor functions are given in Supplementary Note 6. A total of 226 features are generated for each atom in the MOF unit cell.

### Model Architecture

Fig. 2a shows the architecture of our MEPO-ML graph attention network model. The goal of the model is to predict the partial charges of the atoms ${\boldsymbol{Q}}$ from the graph representation of the MOF, $G=\left({\bf{X}},{\mathscr{E}}\right)$, that contains node features ${\bf{X}}\in {{\mathbb{R}}}^{n\times d}$ and edges ${\mathscr{E}}\in {\left\{\mathrm{0,1}\right\}}^{n\times n}$, where $n$ is the number of nodes and $d$ is the number of features for each node. The node features ${\bf{X}}$ are first transformed into hidden node embeddings ${{\bf{H}}}^{0}\in {{\mathbb{R}}}^{n\times h}$ via a multilayer perceptron (MLP), where $h$ is the size of the embedding vector of each node. Then, the initial embeddings ${{\bf{H}}}^{0}$ are processed by an $L$-layer graph attention network (GAT) to generate hidden embeddings ${{\bf{H}}}^{L}\in {{\mathbb{R}}}^{n\times h}$ that describes the local environments of the nodes (more on this later). The hidden local embeddings ${{\bf{H}}}^{L}$ are transformed into the raw charge predictions ${{\bf{Q}}}^{{raw}}\in {{\mathbb{R}}}^{n}$ via another MLP. Charge neutrality is enforced by evenly distributing the excess charges to yield the predicted charges ${{\bf{Q}}}^{{pred}}\in {{\mathbb{R}}}^{n}$ as given in Eq. 11. Unless otherwise specified, the loss is computed based on ${{\bf{Q}}}^{{pred}}$ (i.e., after charge neutralization). Details about the MLPs are provided in Supplementary Note 7.

11 $${{\bf{Q}}}^{{pred}}={{\bf{Q}}}^{{raw}}-\frac{{\rm{sum}}\left({{\bf{Q}}}^{{raw}}\right)}{n}$$

To transfer inter-atomic information across chemical bonds via the graph of the chemical system, for each node, an $L$-layer GAT is applied to the initial embedding ${{\bf{h}}}^{0}\in {{\mathbb{R}}}^{h}$ to generate the local embedding ${{\bf{h}}}^{L}\in {{\mathbb{R}}}^{h}$. Note that ${\bf{H}}$ is the matrix that includes all node embeddings of the whole graph, ${\bf{h}}$ is the embedding vector of a single node, and $h$ is the size of ${\bf{h}}$. Figure 2b depicts the inner workings of the GAT layer. Each layer generates new hidden embeddings for each node in a three-step manner: attention computation, message passing and update. In the attention computation phase, an initial attention score $e\left({{\bf{h}}}_{i}^{l},{{\bf{h}}}_{j}^{l}\right)$ is computed using Eq. 12 for every bonded atom pair $\left(i,j\right)$; this attention score indicates the importance of the embeddings of the neighbor $j$ to the node $i$.

12 $$e\left({{\bf{h}}}_{i}^{l},{{\bf{h}}}_{j}^{l}\right)={{\bf{a}}}^{\left(l,a\right)}{\rm{LeakyReLU}}\left({{\bf{W}}}^{\left(l,a\right)}\left[{{\bf{h}}}_{i}^{l}\parallel {{\bf{h}}}_{j}^{l}\right]\right)$$

In this work, we used the *GATv2* implementation^53^ of the attention function with a 0.2 negative slope for the leaky ReLU function; the superscript $l$ indicates the $l$-th GAT layer $\left(0\le l < L\right)$, ${\bf{a}}$ and ${\bf{W}}$ are both learnable weight matrices, and “$\parallel$” denotes vector concatenation. In each layer, multiple attention scores can be computed for each bond using multiple sets of ${\bf{a}}$ and ${\bf{W}}$; this is called the multi-head attention mechanism, and each set of ${\bf{a}}$ and ${\bf{W}}$ is called an attention head. If $A$ attention scores are computed for each edge in one layer, the superscript $a$ indicates the $a$-th attention head $\left(1\le a\le A\right)$. These attention scores are normalized across all neighbors ${{\mathcal{N}}}_{\left(i\right)}$ using the normalized exponential function (also known as the softmax function) to yield the normalized attention score $\alpha$ (Eq. 13). Self-loops are added to the graph during message passing, therefore ${{\mathscr{N}}}_{\left(i\right)}$ includes all atoms directly bonded to atom $i$ and atom $i$ itself.

13 $${\alpha }_{i,j}^{\left(l,a\right)}=\frac{\exp \left(e\left({{\bf{h}}}_{i}^{l},{{\bf{h}}}_{j}^{l}\right)\right)}{\sum _{k\in\mathcal{N}\left(i\right)}\exp \left(e\left({{\bf{h}}}_{i}^{l},{{\bf{h}}}_{j}^{l}\right)\right)}$$

In the message passing phase, the aggregated message of each node ${{\bf{m}}}_{i}$ is the aggregation of the neighbors’ transformed embeddings (${\bf{W}}{{\bf{h}}}_{k}$) weighted by the attention score ${\alpha }_{i,k}$ (Eq. 14). The aggregation function (AGGR in Eq. 14) can be as simple as a summation, however, in this work it was set as a hyperparameter of the network to be tuned from three functions: sum, mean, and max.

14 $${{\bf{m}}}_{i}^{(l,a)}={\rm{AGGR}}\,(\{{\alpha }_{i,k}^{(l,a)}{{\bf{W}}}^{(l,a)}{{\bf{h}}}_{k}^{l}|k\in {\mathcal{N}}(i)\})$$

To update hidden embeddings as the input for the next GAT layer, the aggregated messages of each node ${{\bf{m}}}_{i}$ from different attention heads are concatenated and then condensed down to a size-$h$ embedding vector via a single feedforward layer; here, a single feedforward layer consists of a single linear layer followed by batch normalization and ReLU activation, denoted as “$\sigma$” in Eq. 15.

15 $${{\bf{h}}}_{i}^{\left(l+1\right)}={\rm{\sigma }}\left(\left[{{\bf{m}}}_{i}^{\left(l,1\right)}\parallel \ldots \parallel {{\bf{m}}}_{i}^{\left(l,A\right)}\right]\right)$$

### Model optimization and training

The graph attention networks (GAT) were constructed with PyTorch Geometric library^41^ and trained using the PyTorch library^54^. Parameters of the GAT were optimized using the AdamW optimizer^55^ with a learning rate of 0.001 and a weight decay coefficient of 0.0005. The GAT was trained with the mean squared error (MSE) loss function and a batch size of 128 graphs. The hyperparameters of the GAT were optimized with Tree-structured Parzen Estimator (TPE) algorithm^56^ using the Optuna library^57^. 100 optimization trials were run using the development set (10% of total data) with 5-fold cross-validation (CV) and the best hyperparameters were picked based on the lowest average MAE from the CV. After that, the GAT with the best hyperparameters were trained on the training set; MSE loss on the development set was computed during training and used as the early stopping criterion to prevent overfitting. The final metrics are computed using the test set.

Three additional models were trained and tested in a similar manner: (1) the gated graph neural network (GGNN) similar to the model purposed by Raza et al.^27^, (2) the random forest regressor (RFR) used by Kancharlapalli et al.^26^, and (3) the gradient boosted decision tree regressor (GBDTR) used by Korolev et al.^25^ These models are trained with the same descriptors for the GAT model; more details on optimizing and training all ML models are described in Supplementary Note 7.

### Atomistic simulations

Grand canonical Monte Carlo (GCMC) simulations were performed to compute the gas adsorption properties of MOFs, using our in-house code available on GitHub (https://github.com/uowoolab/FastMC). For atoms in the MOF structures, the steric and dispersion interactions between gases and MOFs were modeled using the Lennard-Jones potentials from the Universal Force Field (UFF)^58^, where the Lorenz-Berthelot mixing rules were applied for parameters between atoms of different types. Three different fixed partial atomic charge assignment schemes were examined for modeling the guest-host electrostatic interactions: the empirical SQE-MEPO charges^21^, the DFT-derived REPEAT charges^14^, and the predicted charges from the MEPO-ML model developed in this work. Three gases were introduced as guests in our simulations: CO_2_, N_2_, H_2_S. Force field parameters for CO_2_ were taken from García-Sánchez et al.^59^, H_2_S from Kamath et al.^60^, and N_2_ from Provost^61^. All simulations were performed at 298 K with 50,000 MC cycles for equilibration and production. We note that an MC cycle corresponds to n MC steps where n is the number of guest molecules in the simulation. The selectivities for binary guest simulations were calculated using Eq. 16, where $S$ is the selectivity, $N$ is the gas adsorption uptake, $P$ is the partial pressure of the gas.

16 $${S}_{A/B}=\frac{{N}_{A}}{{P}_{A}}/\frac{{N}_{B}}{{P}_{B}}$$

Density functional theory molecular dynamics (DFT-MD) simulations were performed using VASP version 5.4.4^62^ with the Perdew–Burke–Ernzerhof (PBE) functional^63^ with D3 dispersion corrections^64^ and the projector augmented-wave (PAW) method^65^. A 3 × 3 × 3 Monkhorst–Pack sampling of k-points in the Brillouin zone was used. A constant volume MD simulation was run for 14 ps simulation using a 1 fs time step. All masses were scaled to 1 amu to sample the configuration space more rapidly. A stochastic Anderson thermostat^66^ was applied with a probability of 0.2 set at a temperature of 300 K. The simulation was initiated from the experimental CALF-20 structure and all masses were all rescaled to 1.0 amu to generate varied geometric configurations more rapidly. REPEAT charge calculations^14^ were performed in an identifical manner as in the ARC-MOF database^9^.

## Supplementary information

Supplementary Document (PDF)

Supplementary Table E1 (XLSX)

Supplementary Table E2 (XLSX)

## Data Availability

The MOF structures with REPEAT charges used for developing the ML models in this work are obtained from the ARC-MOF (v4) Zenodo repository 10.5281/zenodo.7600474. The training/development/test splits are provided in Supplementary Table E1.

## Code availability

The MEPO-ML charge assignment code with the pre-trained GAT model is available at the GitHub repository https://github.com/uowoolab/MEPO-ML.
