# Supplementary material for: MEPO-ML: a robust graph attention network model for rapid generation of partial atomic charges in metal-organic frameworks
Source: NPJ Comput Mater. 2024 Sep 18;10(1):224. doi: 10.1038/s41524-024-01413-4 (PMC11412901; doi:10.1038/s41524-024-01413-4)
Supplement: Supplementary file 1 — Supplementary Document (PDF) [file 41524_2024_1413_MOESM1_ESM.pdf]

Supplementary Document for

MEPO-ML: A Robust Graph Attention Network Model for Rapid Generation of Partial Atomic Charges in Metal-Organic Frameworks

Jun Luo[a], Omar Ben Said[b], Peigen Xie[b], Marco Gibaldi[a], Jake Burner[a], Cecile Pereira[b,*], Tom K. Woo[a,*]

[a] Department of Chemistry and Biomolecular Science, University of Ottawa, 10 Marie Curie Private, Ottawa K1N 6N5, Canada

[b] TotalEnergies OneTech SE, France

Supplementary Note 1. Figure 3d in the main text provided the charge distribution comparisons of the GAT charges after charge neutralization against the reference REPEAT charges. Here, the comparisons using the GAT raw predicted charges (without charge neutralization) are also provided in **Supplementary Figure 1**.

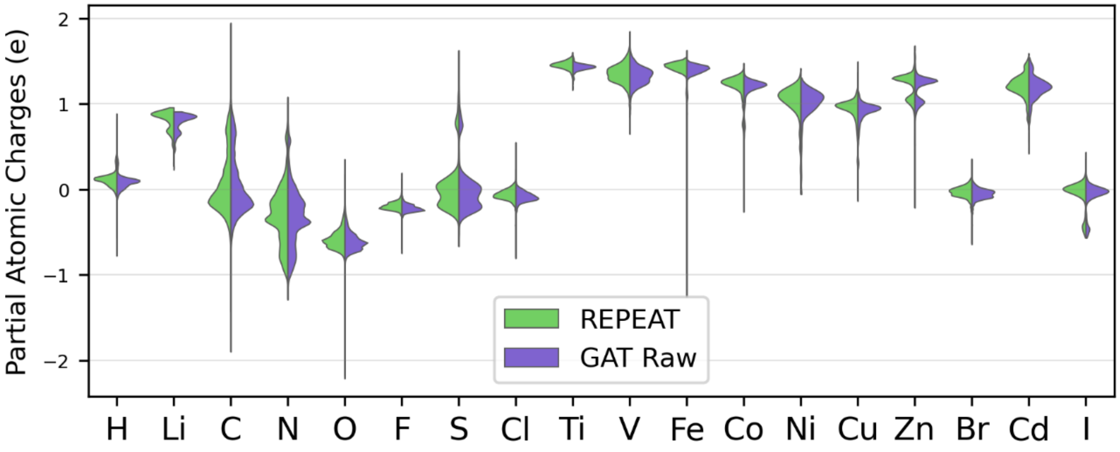

Supplementary Figure 1. **Comparisons of the charge distributions.** A violin plot comparing the distributions of DFT-derived REPEAT charges and the raw charge predictions from the GAT model (without charge neutralization).

Supplementary Note 2. Feature selection analyses

We examined performances of the ML models with reduced feature sets. We computed the correlation coefficient matrix for all 226 features in our "all features" set in the training set. We removed features that have an absolute covariance coefficient of >0.85 with another feature; the feature with lower feature importance will be removed (see Table S4). For the decision tree models (RFR and GDBTR), we are left with 114 features after the feature removal. For the graph neural networks (GGNN and GAT), we further remove all coordination shell descriptors, since these features should be able to be learned from the message passing within the graph; there are 92 features left after the removal. We also investigated feature selection by principal component analysis, where it was performed on all features in the training set; we found that the first 64

principal components were able to recover 99% of the variance. ML models were trained using their optimized hyperparameter, but with different feature sets for performance comparisons, listed in **Supplementary Table 1**.

Supplementary Table 1. MAE performances for ML models from feature selection analyses

ML Model	All Features	Feature Removal Based on Correlation Coefficients	The First 64 Principal Components
RFR	0.0432	0.0457	0.0456
GBDTR	0.0327	0.0332	0.0334
GGNN	0.0322	0.0324	0.0323
GAT (MEPO-ML)	0.0247	0.0251	0.0253

Supplementary Note 3. RAC descriptors and furthest point sampling

During the curation of the ARCMOF database, the revised autocorrelation function (RAC) descriptors were calculated for the diversity analysis across multiple MOF databases. For this work, we directly obtain the RAC descriptors from the Zenodo repository of the ARCMOF database (https://doi.org/10.5281/zenodo.10818822). We selected 20 descriptors each for the metal center and the organic ligand (listed in **Supplementary Table 2**) for selecting diverse subsets of our full training set. Our 1st subset is the 5,638 CoRE MOFs in our training set. For the rest of the MOFs in the training set, we performed furthest point sampling to obtain addition 5,000/10,000/50,000/100,000 MOFs; these samples are added to the 1st subset to create the the 2nd/3rd/4th/5th training sets. To visualize the diversity of these training sets, we reduced the 40 RAC descriptors down to 2-dimensional t-distributed stochastic neighbor embeddings (t-SNEs)[1], plotted in **Supplementary Figure 2**. Note that the furthest point sampling was performed using the 40 RAC descriptors, the t-SNEs were only used for visualization purpose.

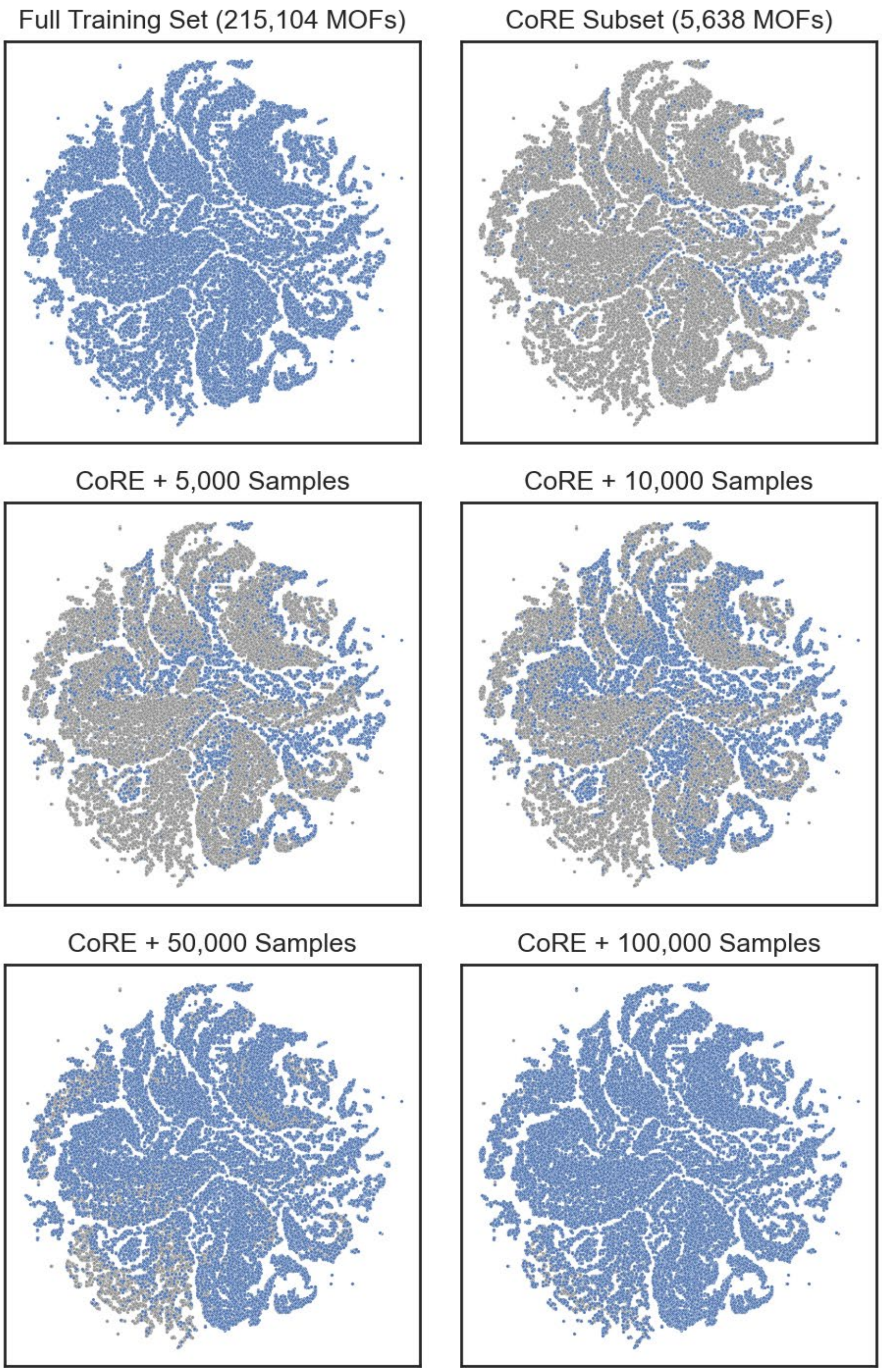

Supplementary Figure 2. Distributions of sampled training sets. MOF chemical distributions of the training sets used to train the machine learning models, visualized in t-distributed stochastic neighbor embedding (t-SNE) plots. Axes and their labels are omitted since the values of the t-SNEs have no chemical meaning.

Supplementary Table 2. Names of the RAC descriptors. For details on naming conventions of these descriptors, please refer to the ARC-MOF publication[2] and the original work from Moosavi et al.[3]

Metal Center Descriptors	Organic Ligand Descriptors
mc-chi-0-all	f-lig-chi-0
mc-chi-1-all	f-lig-chi-1
mc-chi-2-all	f-lig-chi-2
mc-chi-3-all	f-lig-chi-3
mc-Z-0-all	f-lig-Z-0
mc-Z-1-all	f-lig-Z-1
mc-Z-2-all	f-lig-Z-2
mc-Z-3-all	f-lig-Z-3
mc-I-0-all	f-lig-I-0
mc-I-1-all	f-lig-I-1
mc-I-2-all	f-lig-I-2
mc-I-3-all	f-lig-I-3
mc-T-0-all	f-lig-T-0
mc-T-1-all	f-lig-T-1
mc-T-2-all	f-lig-T-2
mc-T-3-all	f-lig-T-3
mc-S-0-all	f-lig-S-0
mc-S-1-all	f-lig-S-1
mc-S-2-all	f-lig-S-2
mc-S-3-all	f-lig-S-3

Supplementary Note 4. Detail accounts of the MOFs with unusual geometric features

We analyzed the 45 MOFs with anomalous MEPO-ML charges and found that 21 of these MOFs contained unusual geometric features, listed in **Supplementary Table 3** below.

Supplementary Table 3. Detail accounts of the MOFs with unusual geometric features

MOF Name in ARC-MOF	Unusual Geometric Features
DB7-ddmof_4897	missing hydrogens from inorganic cluster, odd linker geometry (non-trigonal planar sp^2 carbons in triazine)
DB4-NU-P-4TT-ftw	odd linker geometry (slightly non-linear sp carbons in alkynes) & odd linker structure (X-C≡C-C≡C-X)
DB15-pcu_N77_E79_opt	undercoordinated Ba atom in inorganic cluster
DB12-ZARTAP03_clean	excess H on tetrazole linkers: original publication[4] indicates that only 2 out of every 3 linkers should have 1 protonated N sites; CIF has all tetrazoles protonated
DB12-SOKWUM_clean	missing oxo-t ligands on U (should be UO_2)
DB12-SEWCED_freeONLY	missing H on organic linker
DB12-QISVEU_clean	possible undercoordinated metal atoms
DB12-ORAJEX_clean	disordered K^+ counterion (real position should be between current two atoms), missing oxonium counterions
DB12-HUSQES_clean	missing oxo-t ligands on Mo atoms
DB12-EPONEE_clean	possible undercoordinated metal atoms (removed 4-bromopyridine)
DB12-DUXKUD_clean	missing oxo-t ligands on U (should be UO_2)
DB12-CAJWIV_ion_b	disordered counterions: original publication[5] shows $Li_{1.2}(OH_3^+)_{0.8}$ per unit cell, CIF has 2 Li & 2 OH_3^+
DB10-dia_sym_4_on_6__L_3	single sp^3 carbon atoms as the "metal nodes", also buried atoms
DB1-Uio66Zr-BDC_A-irmof7_A_No259	odd linker (Ph-O-Li groups / undercoordinated Li)
DB0-m9_o19_o23_f0_sra.sym.14	odd linker structure (X-C≡C-C≡C-X & dense -OH functionalization on bicyclooctane)
DB0-m9_o11_o22_f0_sra.sym.41	odd linker structure (dense -COOH functionalization on bicyclooctane)
DB0-m3_o104_o19_f0_fsc.sym.8	odd linker structure (N≡C-C≡N & overbonded S atoms) & odd linker geometry (distorted aromatic rings OR missing hydrogens)
DB0-m2_o36_o36_f0_pts.sym.8	odd linker structure (dense -OH functionalization on tetra-benzene linker)
DB0-m2_o23_o25_f0_nbo.sym.110	odd linker structure (dense -PhF functionalization on bicyclooctane)
DB0-m29_o97_o172_f0_pts.sym.49	potentially missing counterion: Fe(IV) possible but unlikely
DB0-m1_o26_tfs	slightly distorted aromatics

Supplementary Note 5. Definition of coordination shell

We used coordination shells to compute the descriptors that encode chemical bonding environments. For an atom of interest i, the 1st coordination shell includes all atoms that are directly bonded to i, that is, these atoms are 1 bond away from atom i. The 2nd coordination shell therefore includes all atoms that are 2 bonds away from atom i; hence the nth coordination shell includes all atoms that are n bonds away from atom i. An illustration of the first 3 coordination shells is shown in **Supplementary Figure 3** for a 2D graphene fragment; this concept applies similarly to 3D structures and coordination shells that crosses the periodic boundary.

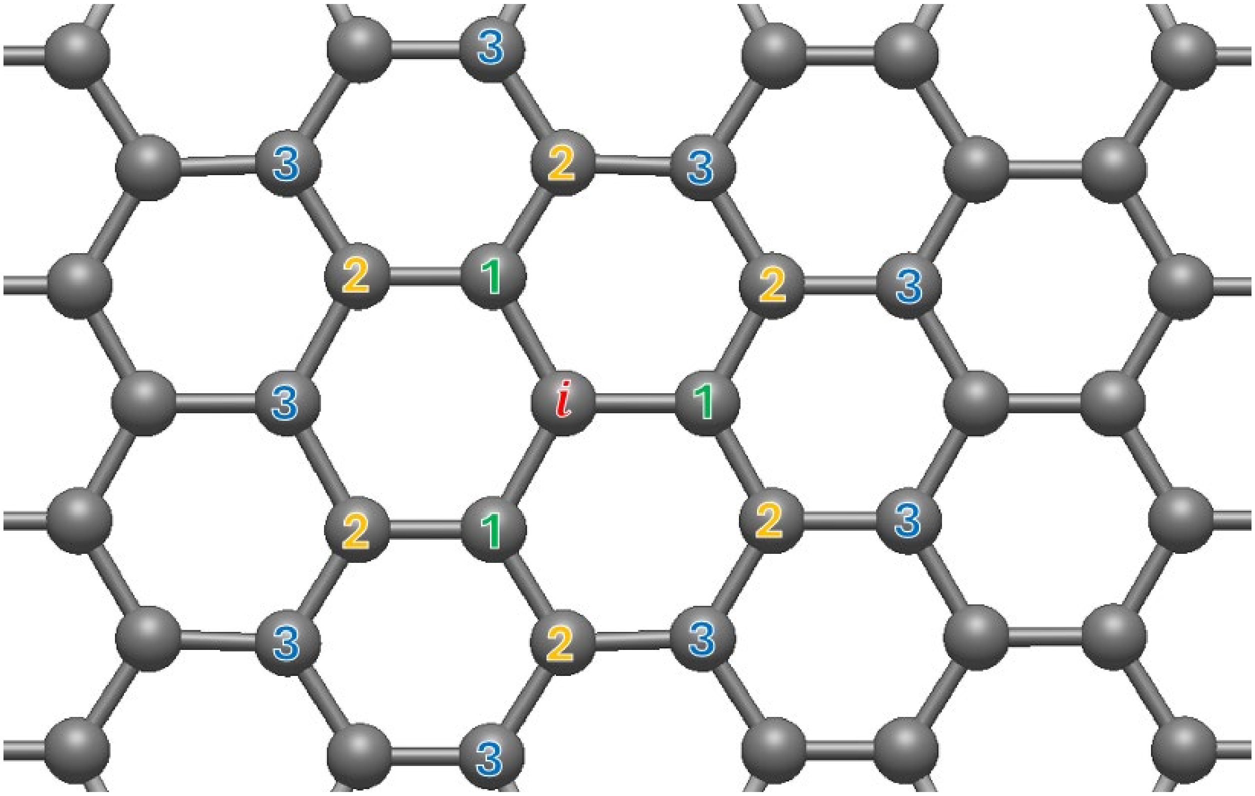

Supplementary Figure 3. Definition coordination shells. The first 3 coordination shells for a 2D graphene fragment: atom of interest i labelled red, atoms in the 1st/2nd/3rd coordination shell labelled green/yellow/blue.

Supplementary Note 6. Parameters for descriptor functions

We used 4 different types of descriptor functions (Eq. 1-10 in the main text) to generate the 226 descriptors as node features for the MOF graphs. These descriptors are generated by combining different parameters for the descriptor functions, list in **Supplementary Table 4**.

Supplementary Table 4. Parameters for descriptor functions

Parameter Name	Symbol	Descriptor Function	Value(s) and Unit(s)
Atomic Property	P	All except Shell*	the Ghosh electronegativity
Minimum Cutoff	r_0	All except Shell*	1.0 Å
Maximum Cutoff	R_{cut}	pwRDF, RWAAP, wRACSF	8.0 Å
		pwADF, AWAAP, wAACSF	6.0 Å
Gaussian Smoothing	α	pwRDF, RWAAP	60.0
	β	pwADF, AWAAP	60.0
	γ	wRACSF	6.0
	η	wAACSF	0.500, 0.170, 0.085, 0.050, 0.034, 0.024, 0.018, 0.014
Radial Bins	R	pwRDF, RWAAP	1.0 to 8.0 with 0.25 Å intervals
	μ	wRACSF	1.0 to 8.0 with 0.50 Å intervals
Angular Bins	Θ	pwADF, AWAAP	0° to 180° with 10° intervals
Angular Phase	λ	wAACSF	± 1

***Note:** The coordination shell descriptors are computed for all 8 atomic properties within the first 4 coordination shell (therefore "cutoffs" does not apply here).

Supplementary Note 7. Hyperparameters for ML models

Hyperparameters of each ML models (listed in **Supplementary Table 5-8**) are optimized using the Optuna[6] library, with 100 trials using the Tree-structured Parzen Estimator (TPE)[7] as the sampler with 20 startup trials; a 5-fold cross-validation (CV) on the development set (10% of total data) was performed in each trial, with the average MAE from the CV as the objective for the hyperparameter optimization. All training of the models were done on the RTX 4090 NVDIA GPU. Random forest regressors (RFR) are trained using the RAPIDS[8] library. Gradient boosted

decision tree regressors (GBDTR) are trained using the CatBoost[9] library, with a maximum of 10,000 iterations and the Bayesian bootstrap type. The graph neural networks (GGNN and GAT) were trained using the PyTorch[10] library and Pytorch Geometric[11] library with the mean squared error (MSE) loss function, the AdamW[12] optimizer, a learning rate of 0.001, a weight decay coefficient of 0.0005, a batch size of 128 graphs. The architecture of the GGNN is the same as the GAT (see Fig 2 in the main text) except the GAT layers are replaced with the GGNN[13] layers. For the graph neural networks (GGNN and GAT), "hid_size" is the size of hidden embeddings during the graph convolutions, "num_conv" is the number of graph convolution layers, "aggr_type" is the type of aggregation function. For the GGNN, "zero_frac" is the fraction of the hidden embedding that were set to zero for the gated graph convolution mechanism. For the GAT, "num_head" is the number of attention heads for the graph attention mechanism. Note that the sizes of the input and output MLPs (see Fig 2 in the main text) are controlled by the "hid_size" hyperparameter. The input MLP is a two-layer MLP with input layer being the size of the node feature vector, the final layer being "hid_size" and the middle (hidden) layer being the average of the two. The output layer is a three-layer MLP, where the input layer being "hid_size", the first hidden layer being the average of "hid_size" and 32, the second hidden layer being 32, and the final layer being 1 as the predicted charge output. Both MLPs are batch normalized and ReLU activated.

Supplementary Table 5. Hyperparameters for random forest regressor (RFR)

Parameters	Values	Best
n_estimators	50, 100, 150, 200	200
bootstrap	True, False	False
max_depth	5, 10, 15, 20	20
max_features	"auto", "sqrt", "log2"	"auto"
min_samples_leaf	1, 2, 3, 4, 5, 6, 7, 8, 9, 10	8
min_samples_split	2, 3, 4, 5, 6, 7, 8, 9, 10	7
accuracy_metric	"r2", "median_ae", "mean_ae", "mse"	"mse"

Supplementary Table 6. Hyperparameters for gradient boosted decision tree regressor (GBDTR)

Parameters	Values	Best
bagging_temperature	0, 1, 2, 3, 4, 5, 6, 7, 8, 9, 10	0
boosting_type	“Ordered”, “Plain”	“Plain”
depth	4, 5, 6, 7, 8, 9, 10	10
l2_leaf_reg	1, 2, 3, 4, 5, 6, 7, 8, 9, 10	1

Supplementary Table 7. Hyperparameters for gated graph neural network (GGNN)

Parameters	Values	Best
hid_size	100, 200, 300, 400, 500, 600, 700, 800, 900, 1000	500
num_conv	1, 2, 3, 4, 5, 6, 7, 8	7
zero_frac	0.1, 0.2, 0.3, 0.4, 0.5	0.1
aggr_type	“sum”, “mean”, “max”	“max”

Supplementary Table 8. Hyperparameters for graph attention neural network (GAT)

Parameters	Values	Best
hid_size	100, 200, 300, 400, 500, 600, 700, 800, 900, 1000	700
num_conv	1, 2, 3, 4, 5, 6, 7, 8	7
num_head	1, 2, 4, 5, 10, 20, 25	20
aggr_type	“sum”, “mean”, “max”	“sum”

References

(1) Van Der Maaten, L.; Hinton, G. Visualizing Data Using T-SNE. *Journal of Machine Learning Research* **2008**, *9*, 2579–2625.

(2) Burner, J.; Luo, J.; White, A.; Mirmiran, A.; Kwon, O.; Boyd, P. G.; Maley, S.; Gibaldi, M.; Simrod, S.; Ogden, V.; Woo, T. K. ARC–MOF: A Diverse Database of Metal-Organic Frameworks with DFT-Derived Partial Atomic Charges and Descriptors for Machine Learning. *Chemistry of Materials* **2023**, *35* (3), 900–916. https://doi.org/10.1021/acs.chemmater.2c02485.

(3) Moosavi, S. M.; Nandy, A.; Jablonka, K. M.; Ongari, D.; Janet, J. P.; Boyd, P. G.; Lee, Y.; Smit, B.; Kulik, H. J. Understanding the Diversity of the Metal-Organic Framework Ecosystem. *Nat Commun* **2020**, *11* (1), 4068. https://doi.org/10.1038/s41467-020-17755-8.

(4) Yan, Z.; Li, M.; Gao, H. L.; Huang, X. C.; Li, D. High-Spin versus Spin-Crossover versus Low-Spin: Geometry Intervention in Cooperativity in a 3D Polymorphic Iron(II)–Tetrazole MOFs System. *Chemical Communications* **2012**, *48* (33), 3960–3962. https://doi.org/10.1039/C2CC18140A.

(5) Yang, S.; Martin, G. S. B.; Titman, J. J.; Blake, A. J.; Allan, D. R.; Champness, N. R.; Schröder, M. Pore with Gate: Enhancement of the Isosteric Heat of Adsorption of Dihydrogen via Postsynthetic Cation Exchange in Metal-Organic Frameworks. *Inorg Chem* **2011**, *50* (19), 9374–9384. https://doi.org/10.1021/IC200967B/SUPPL_FILE/IC200967B_SI_003.PDF.

(6) Akiba, T.; Sano, S.; Yanase, T.; Ohta, T.; Koyama, M. Optuna: A Next-Generation Hyperparameter Optimization Framework. *Proceedings of the ACM SIGKDD International Conference on Knowledge Discovery and Data Mining* **2019**, 2623–2631. https://doi.org/10.1145/3292500.3330701.

(7) Watanabe, S. Tree-Structured Parzen Estimator: Understanding Its Algorithm Components and Their Roles for Better Empirical Performance. **2023**.

(8) Raschka, S.; Patterson, J.; Nolet, C. Machine Learning in Python: Main Developments and Technology Trends in Data Science, Machine Learning, and Artificial Intelligence. *Information (Switzerland)* **2020**, *11* (4). https://doi.org/10.3390/info11040193.

(9) Prokhorenkova, L.; Gusev, G.; Vorobev, A.; Dorogush, A. V.; Gulin, A. CatBoost: Unbiased Boosting with Categorical Features. *Adv Neural Inf Process Syst* **2017**, *2018-December*, 6638–6648.

(10) Paszke, A.; Gross, S.; Massa, F.; Lerer, A.; Bradbury, J.; Chanan, G.; Killeen, T.; Lin, Z.; Gimelshein, N.; Antiga, L.; Desmaison, A.; Köpf, A.; Yang, E.; DeVito, Z.; Raison, M.; Tejani, A.; Chilamkurthy, S.; Steiner, B.; Fang, L.; Bai, J.; Chintala, S. PyTorch: An Imperative Style, High-Performance Deep Learning Library. *Adv Neural Inf Process Syst* **2019**, *32*.

(11) Fey, M.; Lenssen, J. E. Fast Graph Representation Learning with PyTorch Geometric. **2019**.

(12) Loshchilov, I.; Hutter, F. Decoupled Weight Decay Regularization. *7th International Conference on Learning Representations, ICLR 2019* **2017**.

(13) Li, Y.; Zemel, R.; Brockschmidt, M.; Tarlow, D. Gated Graph Sequence Neural Networks. *4th International Conference on Learning Representations, ICLR 2016 - Conference Track Proceedings* **2015**.
